# Statistical Analysis of Large Format Additively Manufactured Polyethylene Terephthalate Glycol with 30% Carbon Fiber Tensile Data

**DOI:** 10.3390/polym16192812

**Published:** 2024-10-04

**Authors:** Katie A. Martin, Jedadiah F. Burroughs, Guillermo A. Riveros

**Affiliations:** 1 Geotechnical and Structures Laboratory (GSL), US Army Corps of Engineers (USACE) Engineer Research and Development Center (ERDC), 3909 Halls Ferry Rd., Vicksburg, MS 39180, USA; katie.a.martin@usace.army.mil (K.A.M.); jedadiah.f.burroughs@usace.army.mil (J.F.B.); 2 Information Technology Laboratory (ITL), US Army Corps of Engineers (USACE) Engineer Research and Development Center (ERDC), 3909 Halls Ferry Rd., Vicksburg, MS 39180, USA

**Keywords:** large-format additive manufacturing (LFAM), additive manufacturing (AM), composites, polyethylene terephthalate glycol (PETG), carbon fiber (CF), ultimate tensile strength, statistical analysis, ANOVA, stress concentration, void space

## Abstract

In large format additive manufacturing (LFAM), a keener understanding of the relationship between the manufacture method and material temperature dependency is needed for the production of large polymer parts. Statistical analyses supported by material properties and a meso-structural understanding of LFAM are applied to elucidate tensile data trends. The data from LFAM polyethylene terephthalate glycol with 30% carbon fiber (CF) (PETG CF30%) panels (diagonal, horizontal, and vertical in the x-y print plane) and injection-molded specimens tensile tested at six different testing temperatures (room temperature, 40 °C, 50 °C, 60 °C, 70 °C, and 80 °C) were used for statistical analyses. A standard deviation, a coefficient of variation, and a two-way and one-way analyses of variance (ANOVA) were conducted. The manufacturing method (44.2%) and temperature (47.4%) have a strong effect on the ultimate tensile strength, in which temperature (82.6%) dominates Young's modulus. To explain the difference between the ultimate tensile strength of vertical, diagonal, and horizontal specimens at room temperature, a visual inspection of the specimen failure was conducted and the maximum stress at the crack tip was calculated analytically. The decreased strength in the diagonal specimens resulted from the reliance on interlaminar adhesion strength. Future work will consider the effect of the void space variation on tensile strength variance.

## 1. Introduction

Additive manufacturing or 3D printing [1], as defined by ISO 17296-3:2104(E), is “a process of joining bulk raw materials to make parts from 3D model data, usually layer upon layer, as opposed to subtractive manufacturing and formative methodologies” [2]. Fused filament fabrication (FFF) [3] or fused deposition modeling (FDM) extrudes a melted plastic layer by layer into a design specified by a sliced CAD file [4] and is the most commonly used type of 3D printing [5]. Large format additive manufacturing (LFAM) [1,6] can print full-scale prototypes that desktop printers with smaller build volumes cannot print [7]. Vicente et al., 2023 define printers according to build volume, which are small- (<1 m^3^), medium- (1–7 m^3^), or high- (>7 m^3^) scale, with medium and high falling under LFAM [1], while Quintana et al., 2022 note that a desktop printer may extrude 1 kg/h and a LFAM printer may extrude 60 kg/h [8]. LFAM often uses pelleted material because filaments result in increased printing times [6]. Fused granular fabrication (FGF) uses thermoplastic pellets instead of a filament [1]. For pellet-fed LFAM, the pellets are compacted, melted, and metered, and a part is built layer by layer [8].

Because polyethylene terephthalate glycol (PETG) [9] has good mechanical properties, such as good durability, high thermal stability and chemical resistance, and good strength and ductility [10], it is commonly used as a 3D printing feedstock material [9,11,12]. Additionally, in an internal naval investigation, PETG did not produce cancerous or damaging fumes when on fire, unlike polylactic acid (PLA) [13] and acrylonitrile–butadiene–styrene (ABS) [13]. Therefore, for the LFAM fabrication of Navy vessels from thermoplastics, PETG is a good option. Carbon fiber (CF) is added to polymers for increased strength [13] and reduced material shrinkage [4], and short-cut carbon fiber is combined with the polymer matrix [14]. Furthermore, PETG [15], PETG with glass fiber [8], PLA with carbon fiber [16], and acrylonitrile styrene acrylate (ASA) with carbon fiber [17] have been investigated in LFAM, making PETG CF a material of interest for further study.

The manufacture method has been shown to affect the strength of the specimens, with injection-molded specimens being stronger than FFF-printed specimens [18]. Strength in FFF-printed parts relies on base material properties, inter-bead bond strength, and void content [19]. Bonding between layers does not achieve full material strength because polymer entanglements are limited by the rapid cooling of beads [11,20,21,22]. Additionally, the void space results in a reduction in part strength because the voids introduce stress concentrations into the part [18,22] which results in a reduced cross-sectional area [16,22]. The size and distribution of the void space are affected by the print parameters, such as the print bed temperature, the nozzle temperature, the layer height, the extrusion multiplier, and the infill density and pattern [16,18,23], with the void space known to vary within a print in both desktop FFF and FGF LFAM [24,25].

Anisotropy is created during desktop FFF and FGF LFAM printing processes. At the same print parameters, prints have greater strength in the direction of the printed bead when compared to the interlaminar strength in the z-direction [15,16,17,23,26]. Additionally, the printed bead has greater strength than the bonding between beads on the same layer [22]. Furthermore, the manufacturing of a carbon fiber polymer in injection molding, desktop FFF, and FGF LFAM results in anisotropy due to fiber alignment during manufacture [8,16,17,27]. In short fiber polymer composite injection-molded specimens, high fiber orientation occurs in thin sections at high shear rates, with the shear orienting the fiber in the direction of flow. The fiber orientation creates anisotropy within the specimen, with the highest tensile strength occurring when force is applied in the direction of the fiber. Processing conditions have a large effect on the final properties of fiber/polymer injection-molded samples [27]. Fiber alignment also occurs in FFF and FGF LFAM printing. During the FFF printing process, carbon fiber alignment results in anisotropy [14]. Quintana et al., 2022 concluded that fiber tended to orient in the direction of flow in large bead size (layer height of 5.08 mm) [8]. Slattery, McClelland, and Hess 2024 found, at a layer height of 2 mm, that a decreased extrusion multiplier resulted in increased fiber content oriented in the direction of flow, with all tested cases showing an orientation greater than 50% [16]. Pintos et al., 2024 [17] found that shorter fibers tended to misorient more than longer carbon fibers and had images showing fibers oriented in the direction of printing. Additionally, carbon fiber is reported to limit the strength of interlayer adhesion and may serve as a crack initiator.

A decrease in Young’s modulus (E) is expected above the glass transition temperature (T_g_) [4]. When a polymer is in the glassy state (i.e., below T_g_), polymer chains are rigid. Above T_g_, the polymer enters the rubber–elastic region, allowing the polymer chains to rotate more freely [28,29]. The increased motion of the polymer chains is a result of the increased temperature, which results in a more flexible material [30]. As the temperature is increased further, the filled and unfilled amorphous polymer’s moduli will begin to converge as the melt condition is approached [4]. PETG is a linear, amorphous polymer [10]. The reported T_g_ of polyethylene terephthalate glycol (PETG) with 30% carbon fiber (CF) (PETG CF30%) is ~65 °C on the DSC and ~82 °C on the DMA [25]. Thermogravimetric analysis is used to determine the carbon fiber content of reinforced polymer composites [25,31,32], and in air, the mass loss of carbon fiber occurs at temperatures 430 °C and 450 °C [31,32], implying the structure of carbon fiber is unlikely to be affected by temperatures near the T_g_ of PETG CF30%.

Substantial work has investigated the tensile properties of desktop FFF, considering the effect of infill pattern [33,34,35], infill percentage [33,34], and material choice [9,33]. The difference in tensile strength between injection-molded and FFF-produced specimens has also been studied [35]. Ghorbani et al., 2022 considered the effect of the extrusion multiplier on tensile strength and dimensional accuracy for desktop FFF ABS parts and found that the void space size and shape affected the final strength of a part [23]. Kichloo et al., 2022 investigated the effect of layer height, infill pattern, infill percentage, and material (PETG or PETG with 20 wt% CF) on tensile strength, flexural strength, and the coefficient of the friction of desktop FFF-printed specimens [33]. Ramírez-Revilla et al., 2022 investigated the tensile properties of desktop FFF-printed polycarbonate (PC), ASA, PLA, and PETG before and after their exposure to a simulated marine environment [9]. Akhoundi and Behravesh [34] studied the effect of infill pattern and infill percentage on tensile and flexural strength and tensile and flexural modulus for PLA specimens printed with desktop FFF [34]. Hsueh et al., 2021 considered the tensile, compressive, and flexural strength and modulus at different printing temperatures and printing speeds for PLA and PETG printed on a desktop FFF printer [36]. Santana et al., 2018 [35] compared the tensile strength and Young’s modulus PLA and PETG specimens printed by desktop FFF-printing with two different infills and two different print planes. Santana et al. [35] 2018 also compared the 3D-printed specimens to injection-molded specimens.

ANOVA has been used to determine the relationship between desired properties. Santana et al. [35] 2018 conducted an ANOVA and Tukey analysis on tensile data. Tezerjani, Yazdi, and Hosseinzadeh 2022 used an ANOVA analysis to determine the effect of print parameters on the cone height of a 4D-printed circular disc after activation [37]. Elkaseer, Schneider, and Scholz 2020 printed PLA using a desktop FFF printer and investigated the effect of printing temperature, printing speed, layer thickness, surface inclination angle, and infill percentage on part surface roughness, energy consumption, productivity, and dimensional accuracy. The Taguchi orthogonal array design of experiments was used to develop the test matrix, and ANOVA with statistical analysis was used to understand the print parameters needed to achieve the desired results [38].

Additionally, other work has considered the tensile strength of LFAM-printed parts.

Castelló-Pedrero et al. [39] 2024 studied the effect of print layer time on the interlayer adhesion strength of ABS with 20% glass fiber. Panels were printed in the XZ direction with a layer height of 1.5 mm, and specimens were water-cut from the panels. Tensile strength and Young’s modulus were reported. Pintos, León, and Molina 2024 [15] ran tensile testing on three different variations of polyethylene terephthalate (PET), referred to as PET1, PET2, and PET3, and one type of PETG printed by LFAM with a 1 mm layer height in the XY and XZ planes. The results were compared to injection-molded specimens, where LFAM PET3 in the XY plane had the highest tensile strength of all the tested materials and manufacture methods and had an XY plane with Young’s modulus that was higher when compared to injection-molded PET3. Sánchez et al., 2020 investigated the tensile behavior of injection-molded and LFAM ASA and ASA CF20% in the X and Z direction (layer height 2.5 mm) and found (1) injection-molded samples of the same material had higher ultimate tensile strength than the specimen printed in the X or Z direction and (2) for ASA CF20% specimens printing in the X direction resulted in a greater Young’s modulus and greater tensile strength than printing in the Z direction [40]. Pintos et al., 2024 [17] considered the effect of the length distribution of carbon fiber within LFAM-printed ASA on tensile strength in the XY and XZ plane. Panels in the XY and the XZ direction were printed with 1 mm layer height in ASA and ASA/CF23%, with one type of CF having shorter lengths and one type having longer lengths. ASA and the short CF ASA had similar tensile strength and Young’s modulus for injection-molded and LFAM specimens printed in the XY plane, though the ASA with longer carbon fibers LFAM-printed in the XY plane exhibited less tensile strength than the injection-molded specimens. Slattery et al., 2024 [16] investigated the effect of the extrusion multiplier (ratio of extruded bead width to tool-path predicted bead width) on tensile strength for PLA, with 10% CF printed with a layer height of 2 mm. The specimens were tested parallel to the printed bead, with the specimens cut from the x-y plane, and perpendicular to the bead, with the specimens cut from a z-plane, where z is the print direction. The tensile strength increased with the increasing extrusion multiplier for the perpendicular testing, while the extrusion multiplier had a negligible effect on the tensile strength for parallel testing. The testing temperature was not varied, and ANOVA was not conducted in the above LFAM studies.

The authors previously conducted a suite of thermomechanical testing for LFAM PETG CF30%, which included the tensile testing of Type V tensile specimens (ASTM D638-14) water-jetted from LFAM panels, printed in the x-y plane with a 2 mm layer height in three different orientations (diagonal, horizontal, and vertical), tested at different temperatures (room temperature, 40 °C, 50 °C, 60 °C, 70 °C, 80 °C). The tensile data showed that injection-molded specimens had the greatest average Young’s modulus and ultimate tensile strength at room temperature, while the ultimate tensile strength and Young’s modulus generally decreased as the temperature increased. The 3D-printed specimens exhibited a relatively stable Young’s modulus and ultimate tensile strength until approximately the glass transition temperature (T_g_), where both Young’s modulus and the ultimate tensile strength decreased. The average data also showed that the vertical average had a greater Young’s modulus and ultimate tensile strength than the horizontal average, and the horizontal average had a greater Young’s modulus and ultimate tensile strength than the diagonal specimens. By 80 °C, the Young’s modulus average appeared to be the same and the ultimate tensile strength of all values were very similar [25].

While tensile data are commonly reported in the literature, understanding the data from a statistical viewpoint highlights the trends seen in material properties. To develop a deeper understanding of the tensile results, the authors have conducted a comprehensive statistical analysis of the data and then applied an understanding of materials behavior and LFAM meso-structure to explain statistical trends. First, the standard deviation and coefficient of variation were calculated for each combination of the testing temperature and the manufacture method (including the sub-category of direction for LFAM-printed specimens). Then, two-way ANOVA was conducted for the testing temperature and the manufacture method (including the sub-category of direction for LFAM-printed specimens) for both Young’s modulus and the ultimate tensile strength. A series of one-way ANOVAs were conducted for both the ultimate tensile strength and Young’s modulus, blocking by manufacture method and by temperature. The results were interpreted and tied back to the material properties, LFAM meso-structure, and stress concentrations from the void space. Understanding the statistical impact of the manufacture method (including the direction for 3D-printed specimens) and testing temperature on tensile strength and Young’s modulus is important for the future application of LFAM parts. Additionally, the effect of specimen direction must be understood to accurately use and interpret results from LFAM test specimens.

## 2. Materials and Methods

Complete information on specimen printing, specimen creation, and specimen testing can be found in Martin et al., 2024, but a general description has been included below for reader clarity [25].

### 2.1. The High Output Research Printer (THOR)

**Figure 1:**
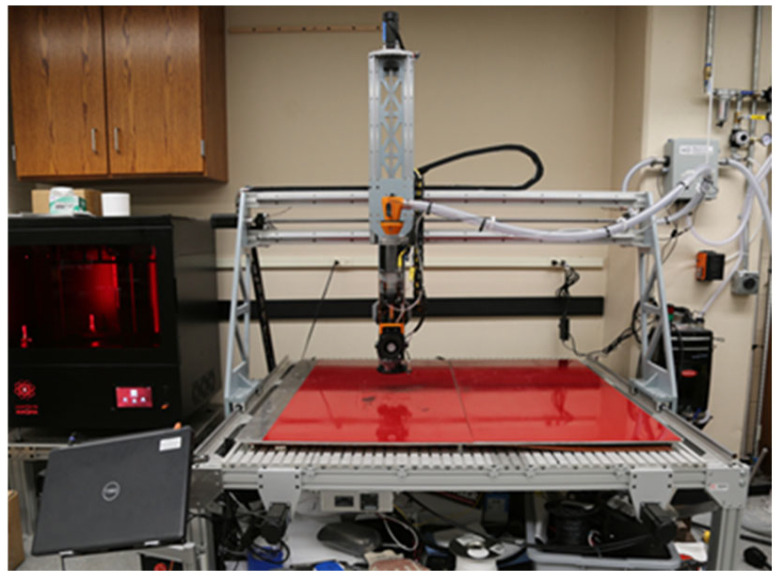
The High Output Research Printer (THOR). Image from Martin et al., 2024 [25] and reproduced with permission from Martin, Polymers; published by MDPI, 2024.

A modifiable LFAM FGF 3D printer, referred to as the High Output Research Printer (THOR) and shown in Figure 1, was developed in-house by the Geotechnical and Structures Laboratory at the US Army Corps of Engineers (USACE) Engineer Research and Development Center (ERDC) in Vicksburg, MI, USA. THOR has an approximate build area of approximately 1.2192 m × 1.2192 mm × 0.508 mm (48″ × 48″ × 20″). The printer operating system is Mach4 software (version 4.2.0.4612). A large print bed model was developed for the Prusa Slicer (version 2.6.1) and is used for all print slicing. Two PID controllers are used to control the nozzle temperature and the print bed temperature separately from the slicer-generated GCODE. The main body was modified from a Millright CNC Power Route XL Assembled (Millright, Leesburg, GA, USA) machine. The extruder system is a MDPH2 extruder (Massive Dimension, Barre, VT, USA) [25]. THOR has a slightly uneven print bed. A MD Feedstock Agitator (Massive Dimension, Barre, VT, USA) uses vibration to ensure consistent pellet flow into the screw and barrel.

### 2.2. Print Material

The printing material was Electrafil PETG 1711 3DP (PETG CF30%) from Techmer PM Polymer Modifiers (Techmer PM, Clinton, TN, USA). PETG CF30% was obtained in pelletized form. A Dri-Air Model HPD-4 RH-150 Drier (Dri-Air Industries, East Windsor, CT, USA) dried pellets for approximately 6 h at 60–66 °C (140–150 °F) [25].

### 2.3. Panel Printing and Tensile Specimen Creation

Panels (355.6 mm × 355.6 mm × 8 mm) were printed with parameters optimized for visually smooth beads. The print parameters are shown in Table 1.

**Figure 2:**
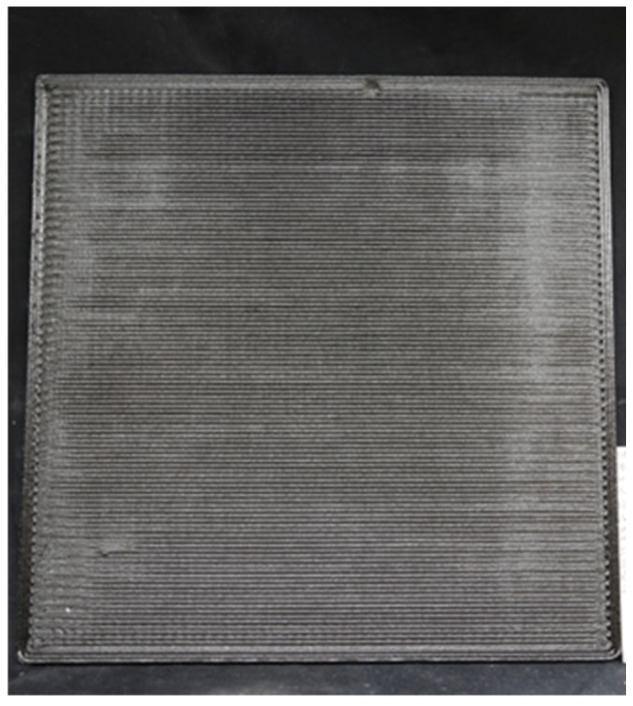
Panel I (355.6 mm × 355.6 mm × 8 mm). Image from Martin et al., 2024 [25] and reproduced with slight crop with permission from Martin, Polymers; published by MDPI, 2024.

Figure 2 shows a printed panel without further post-processing. The panels were decked to a 4 mm thickness. The 4 mm thickness allowed for the equivalent of 2 printed layers at a layer of 2 mm to be tested. Then, tensile specimens were cut in three directions (diagonal, horizontal, and vertical) using Barton garnet 80HPA abrasive (Glens Falls, New York, NY, USA) on a OMAX 5500 water jet (OMAX, Kent, Washington, DC, USA) [25].

**Figure 3:**
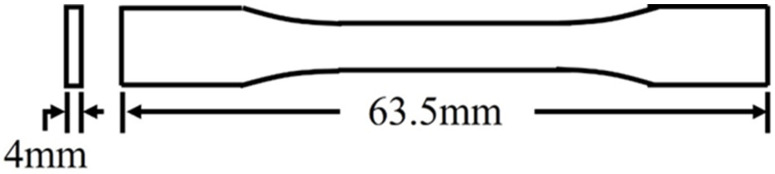
Tensile specimen size and shape from ASTM D638-14 [41]. Image from Martin et al., 2024 [25] and reproduced with permission from Martin, Polymers; published by MDPI, 2024.

The approximate specimen shape is shown in Figure 3. The specimen dimensions were taken in accordance with ASTM D638-14 Type V [41]. Instead of the 3.2 mm thickness [41], a thickness of 4 mm was selected to allow study of two printed bead thicknesses [25].

**Figure 4:**
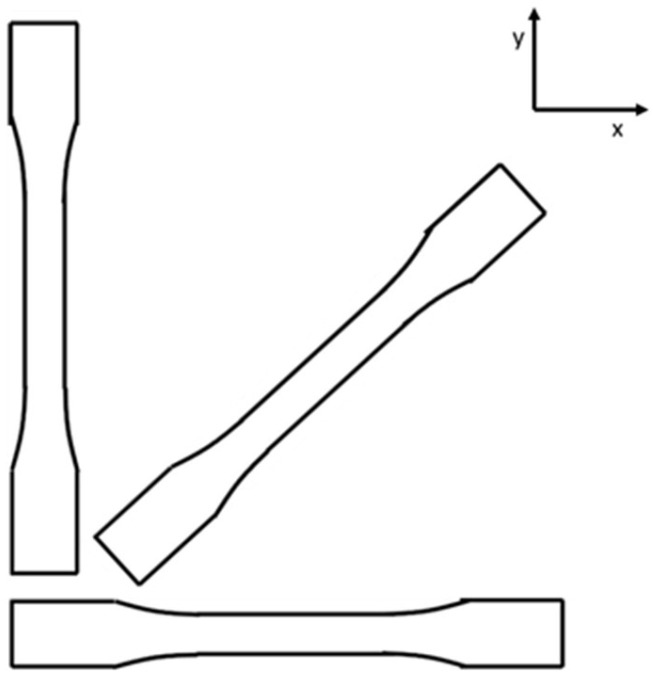
The representative orientation of tensile specimens in the x-y plane in the diagonal, horizontal, and vertical direction, with the z-direction coming out of the page.

The tensile specimens were taken from the x-y plane at 0°, 45°, and 90°, where the z-direction is the build direction upward and the x-y plane is the layer where the beads are printed side-by-side. Figure 4 shows the tensile specimens in the diagonal, horizontal, and vertical direction in the x-y plane, with the z-direction coming out of the page. The specimens were not printed in the shown orientation; rather, a panel was printed, and specimens were cut from the panel in the three selected orientations after printing had been completed. All the specimens were taken from panels printed flat on the print bed.

Table 1 lists the infill pattern as an alternating print pattern of 0°/90°. With a nominal layer height of 2 mm and a specimen thickness of 4 mm, each specimen contains the equivalent of one layer in the 0° direction and one layer in the 90° direction, which would allow strength in both the vertical and horizontal direction and would help mitigate some of the anisotropy that would be seen if the panel had only been printed in the 0° direction [25].

### 2.4. Injection-Molded Tensile Specimens

PETG CF30% was also injection molded for comparison. The mold temperature was 40 °C, and the injector temperature was 250 °C. The pressure was held for 60 s at 350 bar. The specimens were made with a Thermo Scientific MiniJet Pro (Thermo Fischer Scientific, Waltham, MA, USA) [25]. The injection-molded samples were considered fully solid.

### 2.5. Tensile Testing

The tensile testing of the PETG CF30% injection-molded specimens and the specimens taken from the LFAM panels in three directions (diagonal, horizontal, and vertical) was conducted on an Instron Electropuls E3000 (Instron, Norwood, MA, USA) with an environmental chamber for temperature control. The tests were conducted at a displacement rate of 2 mm/min and a range of testing temperatures: room temperature (RT), 40, 50, 60, 70, and 80 °C. The specimens for all the test temperatures were allowed to acclimate for 10 min before testing. Bluehill Universal software (version 4.25) was used to record and analyze Young’s modulus and the ultimate tensile strength. For each test temperature, at least four specimens were tested [25]. Room temperature was approximately 23 °C.

### 2.6. Photography and Microscopy

Images of specimen failure locations were taken with a Canon EOS 5D Mark III (Canon, Ota City, Tokyo, Japan) with a 24–105 mm Canon zoom lens (Canon, Ota City, Tokyo, Japan). Images of the specimen void variance and the failure location were taken with a Zeiss SteREO Discovery V20 microscope (ZEISS, Oberkochen, Germany) and analyzed using Zeiss AxioVision Version 6.1.7601 Build 7601 AxisVs40 V4.8.2.0 (ZEISS, Oberkochen, Germany). The images were taken after the tensile samples were tested.

### 2.7. Statistical Analysis

To better understand the variance and statistical significance of the results for injection-molded specimens and 3D-printed specimens taken in three directions (diagonal, horizontal, and vertical), statistical analyses were performed on the data for Young’s modulus and the ultimate tensile strength. To explain the material behavior and trends of PETG CF30%, a T_g_ of ~65 °C on the DSC and ~82 °C on the DMA was used [25].

#### 2.7.1. Mean, Standard Deviation, and Coefficient of Variation

The average was calculated for each manufacturing method at each testing temperature for the total number of specimens tested (i.e., 4 or 5, as per Martin et al., 2024 [25]). Then, the standard deviation was calculated in Excel (Microsoft, Redmond, WA, USA), using the standard deviation function STDEV. The coefficient of variation was calculated using Equation (1) with the result reported as a percentage.

(1) $$\mathrm{COV}=\frac{s}{a}\times 100\%$$

where

COV = coefficient of variation;

s = standard deviation;

a = average.

#### 2.7.2. ANOVA and Tukey

Four to five specimens, as per Martin et al., 2024 [25], were produced for each temperature and variation (injection-molded, diagonal, horizontal, vertical). To perform ANOVA, four specimens from each set were selected at random if the set had greater than four values, with the remaining datum not considered in the statistical analysis.

The statistical analysis was broken into two main categories, Young’s modulus and the ultimate tensile strength. For each main category, multiple ANOVAs were conducted. First, two-way ANOVA was conducted to consider the combined effects of temperature and manufacture method (including sub-category of direction specimen was taken from the 3D-printed panel). ANOVA was performed on the full 4 × 6 factorial with four replicates for each treatment combination. Afterwards, one-way ANOVA considered the effect of testing temperature on each manufacture method and considered the effect of the manufacturing method at each testing temperature. For both one-way analyses, the statistically different means was ascertained using the Tukey HSD (honestly significant different) procedure, which compares the absolute difference in pairs of means to the Tukey test statistic, defined in Equation (2). The results of the one-way ANOVA are presented in tables that outline the statistical groups of significance. The average of each statistically similar group is presented as well.

(2) $$T=q_{\alpha ,k,N-k}\sqrt{\frac{\mathrm{MSE}}{n}}$$

where

T = Tukey test statistic;

q_α,k,N-k_ = studentized range distribution at α, k, and N;

α = confidence limit;

k = number of treatment levels;

N = total number of data points;

MSE = mean squared error from one-way ANOVA;

n = number of replicates in a single treatment level.

**Figure 5:**
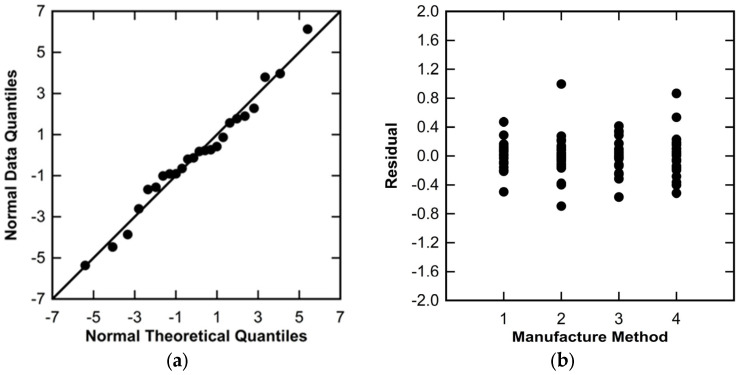
Examples of plots used to verify ANOVA assumptions: (**a**) Q-Q plot for normality; (**b**) residual plot for homogeneity of variance.

**Figure 6:**
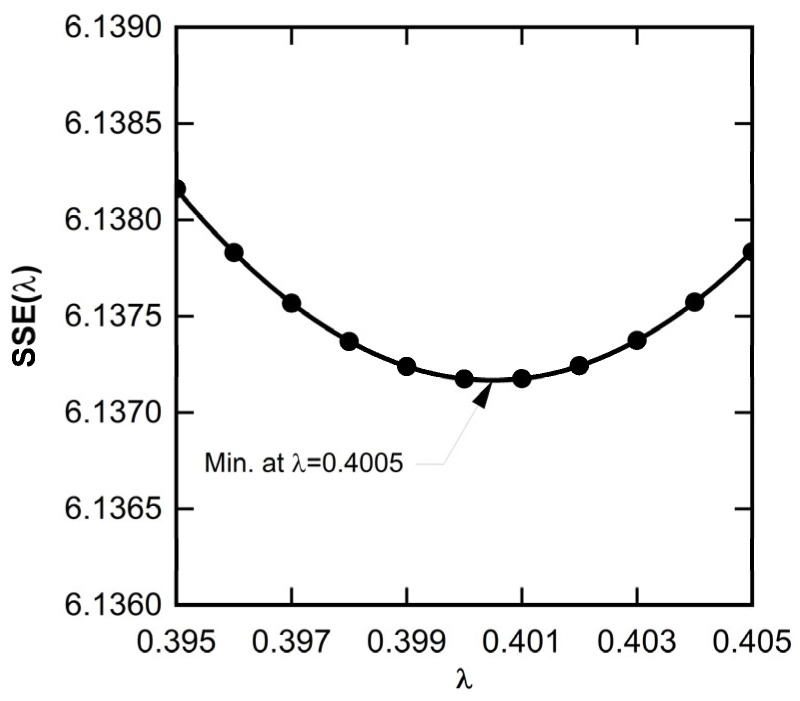
Example of Box–Cox plot to determine optimum transformation parameter by minimizing resulting sums of squares of errors.

Inherent in ANOVA are assumptions of data normality and homogeneity of variance. In this study, these assumptions were verified graphically, using Q-Q plots for normality and residual plots for the homogeneity of variance. Examples of each are shown in Figure 5. To verify the assumption of normality, the data shown in a Q-Q plot should be approximately linear. The assumption of the homogeneity of variance is verified when the magnitude of the observed scatter in data is approximately equal within each treatment factor. The examples shown in Figure 5 would both meet the assumptions for ANOVA. In cases when these assumptions were violated, data transformations were considered using the Box–Cox Method. The Box–Cox method optimizes data analysis by minimizing the sums of squares of error (SSE) term in ANOVA, using power law transformations. Data are transformed using Equation (3), and ANOVA is repeated. A plot of SSE versus the transformation parameter (λ) is developed, with λ corresponding to the minimum SSE chosen as the optimal transformation parameter. An example Box–Cox plot is shown in Figure 6. The data sets that were transformed for analysis are delineated in Section 3 and the appendices, including the optimized λ for each case.

(3) $$y^{\left(\lambda \right)}=\left\{\begin{array}{c} \frac{y^{\lambda }-1}{{\lambda \dot{y}}^{\lambda -1}}\;\lambda \neq 0\\ \dot{y}\ln y\;\lambda =0 \end{array}\right.$$

where,

y = raw datum;

$\dot{y}$ = geometric mean of data set.

## 3. Results and Discussion

### 3.1. Discussion of Variance

For the results below, it is important to remember that mechanical properties change around T_g_ [42], and the material behavior changes from rigid to rubbery [13]. A decrease in Young’s modulus is expected above the glass transition temperature [4]. The reported T_g_ of polyethylene terephthalate glycol (PETG) with 30% carbon fiber (CF) (PETG CF30%) is ~65 °C on the DSC and ~82 °C on the DMA [25].

#### 3.1.1. Ultimate Tensile Strength Variance

Table 2 shows the average ultimate tensile strength, standard deviation, coefficient of variation, and number of specimens tested for all the testing temperatures and manufacture methods. With increasing temperature, the average ultimate tensile strength for injection-molded specimens decreased from RT, with an average ultimate tensile strength of 123.39 MPa to 51.96 MPa at 60 °C. Between 60 °C and 70 °C, there was another drop in the ultimate tensile strength, but the drop in tensile strength between 70 °C and 80 °C was sharper than the previous drop. From RT to 60 °C, the average ultimate tensile strength of the horizontal specimens remained between 22 MPa and 27 MPa. Vertical and diagonal specimens exhibited a similar trend from RT to 60 °C, though vertical specimens had higher average strength (between 30 MPa and 38 MPa) and diagonal specimens had lower average strength (between 14 MPa and 20 MPa) than the horizontal specimens. There was a sharp decrease in tensile strength for all specimens at 70 °C which decreased further at 80 °C to the lowest reported strength for each direction of specimen from the 3D print. All specimens exhibited a decrease in tensile strength after T_g_.

The standard deviation is also reported for each set of specimens. For the injection-molded specimens, the standard deviation varied between 2.15 and 11.04, with the highest variance seen at 50 °C and the lowest at 80 °C. The lowest variance was likely caused by specimens having low ultimate tensile strength because of being above T_g_. There did not appear to be a trend in variance for injection-molded specimens. The coefficient of variation indicated that, relative to the mean, there was a higher percentage of variance within the ultimate tensile data for the injection-molded specimens for 70 °C (15%) and 80 °C (22%) than all other injection-molded specimens, with 50 °C exhibiting the third highest (12%). At all other testing temperatures, the variance was less than 7%.

For the horizontal specimens, the highest standard deviation occurred at a testing temperature of 60 °C (9.26) and the lowest standard deviation occurred at the highest testing temperature, 80 °C (0.26). There was no discernable upward or downward trend in the deviation according to testing temperature. Horizontal specimens exhibited a high coefficient of variation at testing temperatures of 60 °C (37.85%), RT (26.67%), and 40 °C (22.38%). All other coefficients of variation were less than 10%, with the lowest at 80 °C (4.85%). There was no specific trend seen with the coefficient of variation for horizontal specimens.

For the vertical specimens, the highest standard deviation was 6.74 at a testing temperature of 40 °C, and the lowest standard deviation was 0.37 at 80 °C. The standard deviation started low at room temperature, peaked at 40 °C, then slowly dropped for 50 °C and 60 °C, and the lowest two standard deviations occurred at 70 °C and 80 °C. The coefficient of variation exhibited a similar trend, where the coefficient of variation was low at room temperature, peaked at a testing temperature of 40 °C, slowly dropped for 50 °C and 60 °C, and exhibited less variance at 70 °C and 80 °C, though the lowest coefficient of variation was at RT.

For diagonal specimens, the highest standard deviation occurred at a testing temperature of 40 °C (6.00) and the lowest standard deviation occurred at 80 °C (0.61). Similar to the horizontal standard deviations, the highest deviation occurred at 40 °C, smaller deviations occurred at 50 °C and 60 °C, and the lowest standard deviations occurred at 70 °C and 80 °C. All coefficients of variation were above 10%, with the majority above 20%. The highest coefficient of variation occurred for tensile strength at 40°C (31.51%) while the lowest coefficient of variation occurred at 70 °C (10.44%). There did not appear to be a trend in the coefficient of variation data.

The presented data lead to a couple of conclusions. First, after T_g_, there was a significant decrease in tensile strength; this decrease was most noticeable in the 3D-printed specimens after 60 °C and the injection-molded specimens after 70 °C. The injection-molded average tensile strength exhibited a decrease in strength with increasing temperature before T_g_ generally not seen in the 3D-printed specimens. Second, there was variability within the specimens, though there was not a consistent trend across all the temperatures or manufacture methods. The 3D-printed specimens did exhibit their lowest standard deviations for 70 °C and 80 °C (after T_g_ had been reached), and the injection-molded specimens exhibited their lowest standard deviation at 80 °C. The low standard deviation is likely a result of interrelated factors: lower strength and a smaller range of strength values. Above T_g_, there is a decrease in tensile strength, which lessens the effect of anisotropy caused by testing direction and fiber orientation. Similarly, there was no specific trend seen for coefficients of variation. The injection-molded specimens exhibited the highest variation relative to the mean at 70 °C and 80 °C. Of the diagonal specimen variance, the lowest occurred at 70 °C and the middling value occurred at 80 °C. Horizontal and vertical specimens had variances of less than 10% at 70 °C and 80 °C.

The diagonal specimens showed significantly a high coefficient of variation when compared to the other three manufacture methods. While all three other manufacture methods had at least one set of specimens with a coefficient of variation above 20%; they also had at least three coefficients of variation below 10%. Diagonal specimens did not have a single set of specimens with a coefficient of variation of less than 10%, with the majority above 24%. This indicates that the data for diagonal specimens were consistently more variable for each tested temperature than the other manufacture methods.

There is variation within the void space of FGF- and FFF-printed parts [24,25,43], meaning there is variation in the applied stress needed to initiate a crack, resulting in a variation in ultimate tensile strength. The vertical and horizontal specimens only had weaker interlaminar strengths in one layer affected by the applied force, while the diagonal specimens had two layers, which resulted in the variation in the void space having a greater effect on the resulting ultimate tensile strength for diagonal specimens.

Finally, while there was a noticeable trend in temperature and strength around T_g_, there was nothing particularly illuminating about the variance results other than to say that the data were variable within each tested set without a clear trend in variance based on the testing temperature. The coefficients of variation for injection-molded specimens were generally lower than the 3D-printed specimens, with the diagonal higher than all other manufacturing methods.

**Figure 7:**
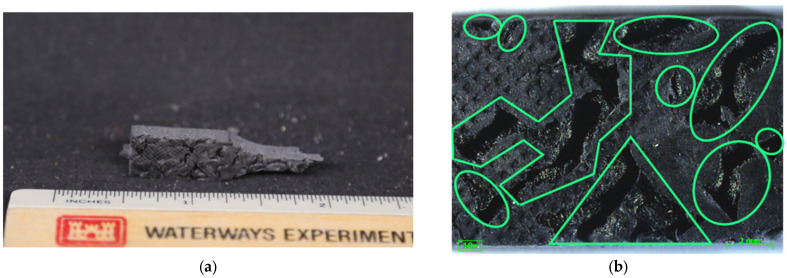
Tested ASTM D638-14 Type V diagonal specimen showing void space variation. Slight dotted pattern on left of sample from tensile testing clamps: (**a**) diagonal sample with variable void space; (**b**) magnified sample showing size and spatial difference in visible void space, with the green indicating void space.

While injection-molded parts are solid, FGF parts are built layer by layer. Variation in the void space in desktop FFF and LFAM [24,25] and variation between FFF-printed parts of the same type and across a print [43] has been reported in the literature. Figure 7 shows the variation in the void space location and size within the grip section of a tested diagonal tensile specimen, with the green in Figure 7b indicating the void space. Additional images of the specimen void space variation can be seen across different specimens in Appendix C.

The void space in FFF prints can serve as stress concentrations [18]. In the calculation of the applied stress of an elliptical hole, when holding the applied stress constant, increasing the size of the hole increases the stress felt at the hole tip. Stress concentrations generally have a greater impact on brittle materials [13]. If the void space serves as a location of stress concentration, a larger length (i.e., larger void) results in greater stress applied along the tip of a crack. As the void space is variable within printed specimens, it implies that there will be a variance of strength related to the variance of the void space and void size within a print. Additionally, the void space occurs between printed beads [25] where the interlaminar strength is known to be weaker than the strength perpendicular to the printed bead [15,16,17,40], meaning the location of highest potential stress occurs where the material strength is weakest. It follows that (1) injection-molded specimens are expected to be stronger than the FGF-printed specimens and (2) the variation in the void space will cause more variation in ultimate tensile strength in the FGF-printed specimens, which would not be expected to be seen in the injection-molded specimens.

#### 3.1.2. Young’s Modulus Variance

Table 3 shows the average Young’s modulus, standard deviation, coefficient of variation, and specimen number for all testing temperatures and manufacture methods. The injection-molded specimens exhibited the highest average Young’s modulus from RT to 70 °C, with the vertical specimens having the next highest average Young’s modulus. The diagonal and horizontal specimens have similar Young’s moduli, but the horizontal was generally higher for the range of RT to 70 °C. All the specimens exhibited a decrease in the average Young’s modulus from 60 °C to 70 °C, with the vertical showing the sharpest decrease (4.58 GPa to 2.37 GPa) and the injection-molded showing the least absolute decrease (6.85 GPa to 6.05 GPa). All the specimens showed a more significant decrease in the average Young’s modulus from 70 °C to 80 °C, with the injection-molded showing the sharpest decrease (6.05 GPa to 0.10 GPa) and the vertical having the slightest decrease (2.37 GPa to 0.02 GPa). At 80 °C, 3D-printed specimens have an average Young’s modulus of 0.03 or 0.02 GPa, with the injection-molded average at 0.10 GPa. While the effect of the DSC T_g_ of ~65 °C starts to be seen in the 3D-printed specimens, the full effect of the transition is seen for all specimens at 80 °C. The T_g_ from the DMA was previously reported to be ~82 °C [25].

All manufacture methods exhibited their lowest standard deviations at 80 °C; however, both injection-molded and diagonal exhibited their highest coefficient of variation by a significant amount, at 125.33% and 101.91%, respectively. All the specimens at all the temperatures had standard deviations less than 1.0, except for the horizontal at 60 °C (1.20). The injection-molded, horizontal, and diagonal standard deviations did not exhibit a specific trend. The vertical specimens showed increasing standard deviation until 50 °C, where the standard deviation peaked, after which a downward trend continued for the rest of the testing temperatures. At 80 °C, all the 3D-printed specimens had standard deviations at least a factor of ten less than the standard deviations of all the other testing temperatures, likely because above T_g_, a decrease in Young’s modulus occurred that resulted in the effect of the manufacturing method becoming much less significant.

Considering the coefficients of variation, the injection-molded generally increased, with a comparatively high value of the coefficient of variation at 80 °C (range of 2.83% to 125.33%). The horizontal had its highest coefficient of variation at 60 °C (29.17%) and the lowest at RT (4.00%). The coefficient of variation for vertical specimens at all testing temperatures was between 8–16%. Most diagonal specimens had a coefficient of variation less than 7%, the two highest coefficients at 50 °C (14.07%) and 80 °C (101.91%).

Similar to the average tensile strength, a decrease in the average Young’s modulus was seen from 60 °C to 70 °C for 3D-printed specimens. There was a decrease for injection-molded specimens, but it was much less pronounced. Also similar to average tensile strength, a decrease in the average Young’s modulus was seen from 70 °C to 80 °C, which was stark for the injection-molded specimens. Both the average tensile strength and the average Young’s modulus exhibited a decrease after the T_g_ of PETG CF30% had been surpassed, which was expected.

Also similar to the average tensile strength, there was no consistent standard deviation or coefficient of variation trend from RT to 70 °C. At 80 °C, all the specimens exhibited low standard deviations, especially the 3D-printed specimens, while the injection-molded and diagonal exhibited very high coefficients of variation. More work should be conducted to understand the impact of 3D-printing on the extent of specimen variance, especially in comparison to injection-molded specimens. Additionally, more specimens per testing temperature should be run to more accurately understand the expected variance at each testing temperature and manufacture method.

While general trends were visible in the data, the statistical relationship between manufacture methods was not fully elucidated; therefore, two-way and one-way ANOVAs were conducted to create a more complete understanding.

### 3.2. Ultimate Tensile Strength ANOVA

#### 3.2.1. Two-Way ANOVA of Manufacturing Method and Testing Temperature for Ultimate Tensile Strength

Two-way ANOVA allows for the comparison of multiple levels of two different treatments. In this study, the two categories of treatments were manufacture method (to include the sub-category of direction for 3D-printed specimens) and testing temperature. ANOVA was performed on the full 4 × 6 factorial with four replicates for each treatment combination. ANOVA results are presented in Table 4. As is shown, ANOVA indicated that both the manufacturing process and testing temperature were highly significant at a confidence level (α) of 0.01. Additionally, the interaction effect between the manufacturing process and testing temperature was also highly significant. These results suggested with at least 99% confidence that the manufacturing process, testing temperature, and interaction effect all statistically influenced the expected ultimate tensile strength. Literature supports these statistical findings. The manufacture method has been shown to affect strength, with injection-molded specimens being stronger than 3D-printed specimens [18]. Around T_g_, which is ~65 °C for PETG CF30% [25], there is a decrease in Young’s modulus [4], which is reflected by a decrease in ultimate tensile strength.

**Figure 8:**
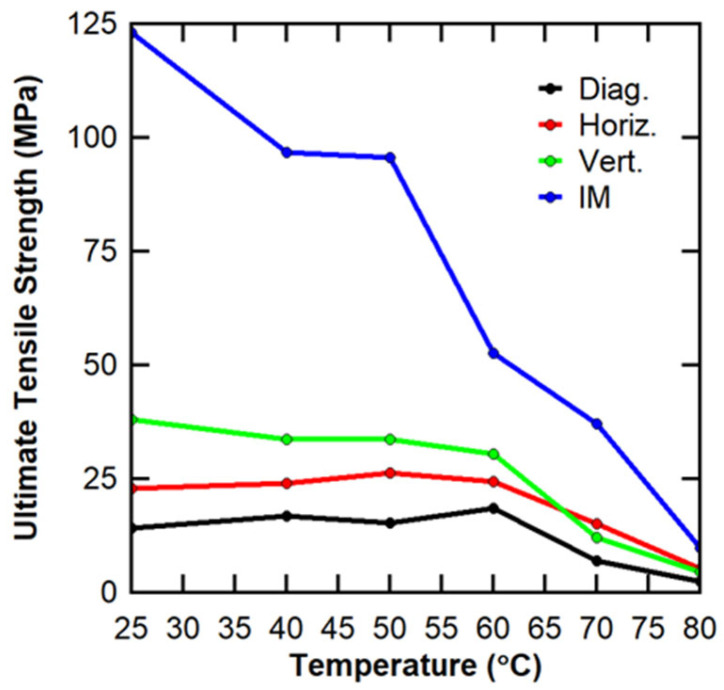
Two-way ANOVA interaction plot for ultimate tensile strength, where IM refers to injection molding, Vert refers to vertical, Horiz refers to horizontal, and Diag refers to diagonal.

Because the interaction effect was shown to be statistically significant, the interpretation of the two-way ANOVA results was not as straightforward as preferred. To determine how critical the interaction effect is, an interaction plot is shown in Figure 8. At testing temperatures at or below 60 °C, the lines did not cross, revealing that the interaction effect was only critical above that temperature. Previous testing by the authors has shown that the glass transition temperature (T_g_) of PETG CF30% is ~65 °C on the DSC and ~80 °C on the DMA [25], indicating that the interaction effect was only critical above the DSC T_g_, where the material switches from rigid to rubbery and exhibits a decrease in tensile strength [13]. However, because lines in the interaction plot did cross within the temperatures of interest, the interaction effect could not be neglected in the overall analysis.

The interaction plot also revealed differences in the behavior over the testing temperatures for the various manufacturing processes and orientations. As expected, the ultimate tensile strength was shown to decrease with the increasing temperature for vertical orientation and for injection molding. The magnitude of the decrease at each temperature step was inconsistent, but a general downward trend was followed. That behavior was not observed with 3D-printed specimens in the diagonal or horizontal orientations. A slight increase in ultimate tensile strength was observed for these orientations up to 60 °C and 50 °C, respectively. It is hypothesized that better bonding was achieved between the printed layers in these orientations as the temperature increased initially. As the temperature neared and/or surpassed the T_g_, a reduction in the strength with increasing temperature was then observed, which is expected. The behavior above T_g_ was consistent regardless of the manufacturing process or specimen orientation.

**Figure 9:**
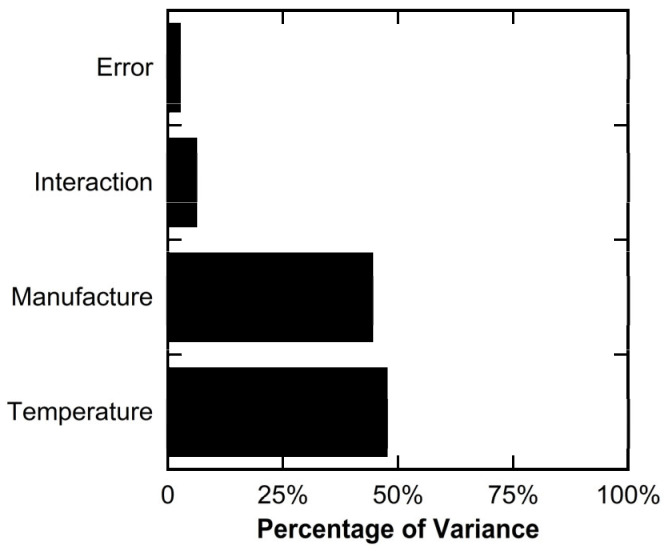
Percentage of variance explained by each treatment factor in two-way ANOVA of ultimate tensile strength data.

By comparing the ratios of the sums of squares for each source of variance, it can be shown that the differences in the manufacturing process explained 44.2% of the total observed variance in the data. The literature notes the effect of the manufacturing process (injection-molded vs. 3D-printed, effect of print parameters) on part strength [18,33,34]. Testing temperature explained 47.4% of the total observed variance, and the interaction effect explained 6.0% of the total observed variance. The remaining 2.4% was explained by the error term that takes into account the variance between replicates. The relative significance of each of these factors is presented graphically in Figure 9. With the error term accounting for such a low percentage of the total variance, the two-way ANOVA had strong statistical power.

#### 3.2.2. Ultimate Tensile Strength One-Way ANOVA: Testing Temperature

To further investigate the statistical differences between the testing temperatures, a series of one-way ANOVAs were performed on various data subsets blocked by the manufacturing process (3D printing—diagonal, 3D printing—horizontal, 3D printing—vertical, injection molding) across six levels of single treatment (testing temperature). The Tukey HSD procedure was used to determine which means were statistically different.

Table 5 shows the one-way ANOVAs for tensile strength blocked by the manufacturing process. For all the manufacturing methods, the measured means for the various temperatures were statistically different at α of 0.01, suggesting 99% confidence that differences existed. For all the 3D-printed specimens, regardless of the direction, there was a statistical difference in the ultimate tensile strength seen between the specimens below T_g_ and above T_g_. The injection-molded specimens generally showed statistical difference in ultimate tensile strength with increasing temperature. When blocking data by the manufacturing process, it was generally shown that the testing temperatures at or above 70 °C significantly influenced the ultimate tensile strength. This is most likely attributable to the testing temperature exceeding the glass transition temperature for PETG CF30% (~65 °C). The tensile strength means, the Tukey mean statistic, and the average difference of means are reported in Appendix A.

#### 3.2.3. Ultimate Tensile Strength One-Way ANOVA: Manufacture Method

Two-way ANOVA revealed that the manufacturing process significantly affected the expected ultimate tensile strength. To further investigate the statistical differences between the manufacturing processes, a series of one-way ANOVAs was performed on various data subsets blocked by testing temperature. These analyses can be thought of as companion analyses to the previous one-way ANOVA series on measured ultimate tensile strengths at various testing temperatures. Four different manufacturing processes/orientations (3D-printed horizontal, 3D-printed vertical, 3D-printed diagonal, and injected-molded) were considered as treatment levels in one-way ANOVA for RT, 40 °C, 50 °C, 60 °C, 70 °C, and 80 °C. As with the temperature analyses presented earlier, the statistically different means can be ascertained using the Tukey HSD procedure.

Table 6 shows the statistically similar groups grouped by manufacture method when compared to tensile strength. The average ultimate tensile strength of the group is reported in parentheses. More specifically, if there is only one group, the average tensile strength is reported; if there are two or more groups, the average of both groups is reported. For room temperature, there were three statistically distinct groups, injection molding (IM), vertical (V) and then diagonal and horizontal (D/H). For 40 °C and 50 °C, the horizontal data were statistically similar to both the diagonal and vertical data. Injection molding remains in its own group. At 60 °C, all three 3D-printing directions were statistically similar, with horizontal and vertical being statistically similar at 70 °C and 80 °C and diagonal being statistically different. Injection molding remained statistically distinct from the 3D-printed specimens for all temperatures. As the temperature increased, the average ultimate tensile for injection molding generally showed a decreasing trend, even before T_g_. Both injection-molded and 3D-printed specimens showed a sudden decrease in ultimate tensile strength between 60 °C and 70 °C, during which the T_g_ was passed. From 70 °C and 80 °C, both injection-molded and 3D-printed specimens exhibited a continued downward trend, though injection molding exhibited a greater absolute decrease in strength.

The data indicated that (1) injection-molded specimens maintained a higher tensile strength than the 3D-printed specimens regardless of selected testing temperature, (2) the T_g_ resulted in the decreased tensile strength for all the manufacture methods, and (3) while there was a statistically significant difference in tensile strength between the diagonal, horizontal, and vertical specimens at room temperature, at higher temperatures, the differences between the horizontal and vertical specimens became statistically insignificant. The higher injection molding strength reflects the literature, where 3D-printed specimens are shown to be generally weaker than their injection-molded comparisons [18] because they have limited interlayer bonding and stress concentrations from the FFF process [25]. A decrease in tensile strength is expected above T_g_ as the PETG CF30% switches from rigid to rubbery [13].

Additional one-way ANOVA, Tukey statistical mean, and average difference tables are available in Appendix A.

### 3.3. Young’s Modulus

#### 3.3.1. Two-Way ANOVA of Manufacturing Method and Testing Temperature for Young’s Modulus

The complete data set for Young’s modulus was analyzed to understand the effects of the manufacturing process and orientation (for 3D prints only) and testing temperature both independently and in conjunction. The same treatment factors considered for the ultimate tensile strength were also considered for Young’s modulus. ANOVA was performed on the full 4x6 factorial with four replicates for each treatment combination. Two-way ANOVA results are presented in Table 7. As was seen with the ultimate tensile strength analysis, two-way ANOVA indicated that both the manufacturing process and the testing temperature were highly significant even when using α of 0.01. Additionally, the interaction effect between the manufacturing process and the testing temperature was also highly significant. These results suggested with at least 99% confidence that the manufacturing process, testing temperature, and interaction effect all statistically influence the expected Young’s modulus.

**Figure 10:**
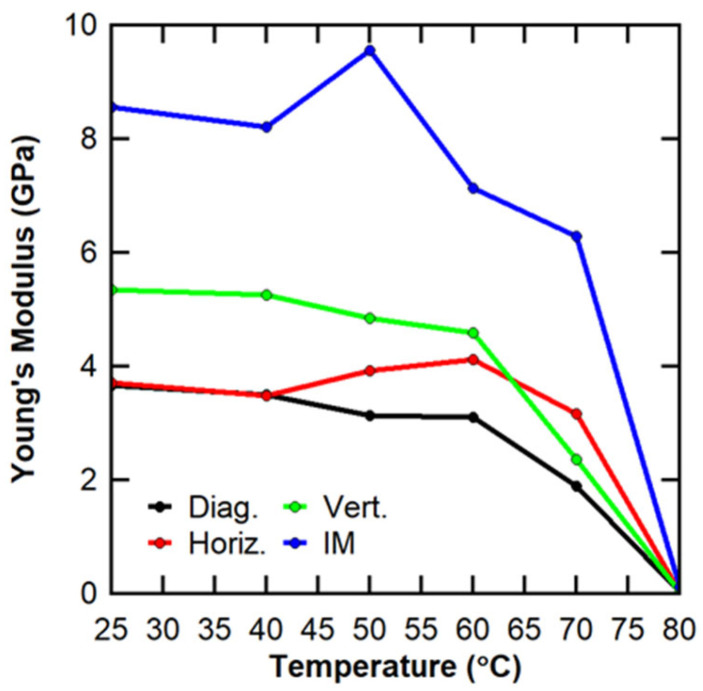
Two-way ANOVA interaction plot for Young’s Modulus, where IM refers to injection molding, Vert refers to vertical, Horiz refers to horizontal, and Diag refers to diagonal.

Because the interaction effect was shown to be statistically significant, the interpretation of the two-way ANOVA results for Young’s modulus was also not straightforward. To determine the importance of the interaction effect, an interaction plot (shown in Figure 10) was developed. Once again, the lines on the interaction plot crossed, so the interaction effect could not be neglected in the overall analysis.

**Figure 11:**
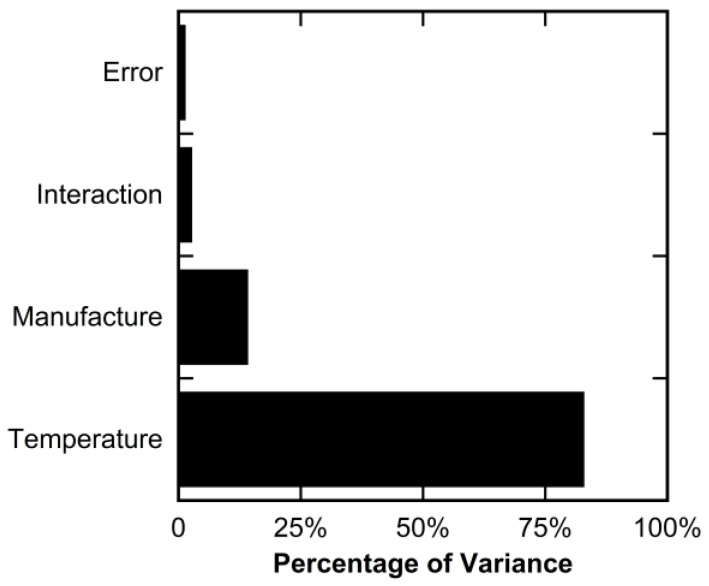
Percentage of variance explained by each treatment factor in two-way ANOVA of Young’s modulus data.

By comparing the ratios of the sums of squares for each source of variance, it can be shown that the testing temperature was the most significant factor, explaining 82.6% of the total observed variance in the data. The manufacturing process only explained 13.9% of the total observed variance, and the interaction effect explained 2.4% of the total observed variance. This interaction effect was less significant for Young’s modulus than for the ultimate tensile strength. The remaining 1.1% was explained by the error term that considers the variance between replicates. The relative significance of each of these factors is presented graphically in Figure 11. With the error term accounting for such a low percentage of the total variance, the two-way ANOVA for Young’s modulus also had strong statistical power.

The findings of the two-way ANOVA aligned with previous research in the literature, where a sudden change in Young’s modulus is expected above T_g_ [13].

#### 3.3.2. Young’s Modulus One-Way ANOVA: Testing Temperature

To dive deeper into the statistical differences between measured Young’s moduli at different testing temperatures, a series of one-way ANOVAs, shown in Table 8, were performed on various data subsets blocked by the manufacturing process (the injection molding and direction taken from the panel for 3D prints). Following the same protocol used for ultimate tensile strength, the statistically different means were identified using the Tukey HSD procedure. For all the manufacturing methods, the measured means for the various temperatures were statistically different at a confidence limit of 0.01, suggesting 99% confidence that differences exist. All manufacture types experienced a decrease in Young’s modulus as the temperature increased past T_g_, which was expected. When blocking data by the manufacturing process, it was generally seen that testing temperatures at or above 70 °C significantly influenced Young’s modulus. The effect was observed for all manufacturing approaches at 80 °C and three manufacturing approaches at 70 °C. This is most likely attributable to the testing temperature exceeding the glass transition temperature for PETG CF30% (~65 °C). Sample means and absolute difference tables are shown in Appendix B.

#### 3.3.3. Young’s Modulus One-Way ANOVA: Manufacture Method

Table 9 shows the statistically similar groups grouped by the manufacture method when comparing Young’s moduli. Similar to the tensile data, the initial data were broken into three statistically significant groups, injection molding with the highest Young’s modulus, vertical with the middle Young’s modulus, and diagonal and horizontal with the lowest values. The injection-molded specimens stayed statistically distinct from the 3D-printed specimens until 80 °C. At 80 °C, all the manufacturing methods had statistically similar data, likely due to the convergence of Young’s modulus above T_g_. For the 3D-printed specimens, the vertical Young’s modulus was distinct from the diagonal and horizontal Young’s modulus for testing at room temperature and 40°; however, at 50 °C, the horizontal Young’s modulus was statistically similar to both the diagonal and vertical while the diagonal and vertical were statistically different. For 60 °C, the 3D-printed specimens were all statistically similar. At 70 °C, the horizontal Young’s modulus was statistically different from the diagonal and vertical Young’s modulus. Note that, while there was a drop in Young’s modulus between 60 °C and 70 °C (T_g_ ≈ 65 °C), it was not as drastic as the drop in the ultimate tensile strength. The injection molding Young’s modulus average was approximately the same at room temperature and 40 °C, peaked at 50 °C and then started to drop thereafter.

The one-way ANOVA, Tukey statistical mean, and average difference tables are available in Appendix A.

### 3.4. The Effect of Print Meso-Structure and Specimen Creation on Ultimate Tensile Strength and Young’s Modulus

One important note to make from the statistical analyses presented is the behavior of the LFAM tensile specimens taken from the panel in three directions (vertical, diagonal, and horizontal). At room temperature for both Young’s modulus and ultimate tensile strength, the vertical orientation was statistically distinct from diagonal and horizontal orientations. As the temperature increased, the statistical difference decreased until, at 60 °C and 80 °C, Young’s modulus for all three directions were statistically similar and at 60 °C, the ultimate tensile strength for all three directions were statistically similar. The diagonal is statistically different at 70 °C and 80 °C for ultimate tensile strength. For the average ultimate tensile strength at room temperature, the vertical orientation was the strongest (37.98 MPa), the horizontal orientation was next (22.89 MPa), and the diagonal orientation was last (14.17 MPa).

Some discussion on the differences is necessary. The panels were printed with an alternating 0°/90° pattern, meaning that each layer was printed perpendicular to the layer below and above it. The specimens were then decked to a thickness of 4 mm, which resulted in having two equivalent printed beads in a specimen. That would mean that for specimens cut from the horizontal and vertical direction, there would be one bead pulled in the direction of the force, implying that the ultimate tensile strength should be approximately the same for vertical and horizontal specimens. There is about a ~15 MPa difference in the average tensile strength at room temperature between the vertical and horizontal; meanwhile, the diagonal specimens have the least strength of all tested directions.

**Figure 12:**
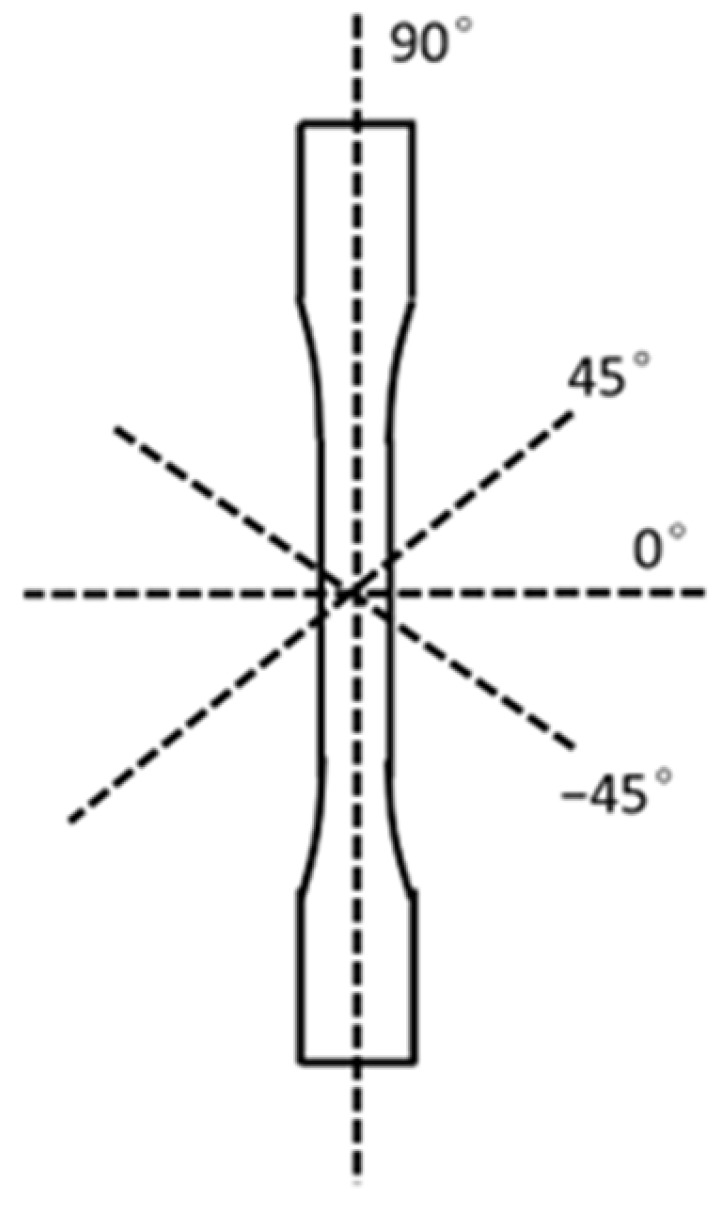
ASTM D638-14 Type V specimen with all possible directions of print layers denoted (90°, 45°, 0°, and −45°).

While there was an expectation of two full beads, the reality was more nuanced. Figure 12 demonstrates all possible bead directions (90°, 45°, 0°, −45°). The images of the samples in the diagonal, horizontal, and vertical orientation can be seen in Appendix C.

**Figure 13:**
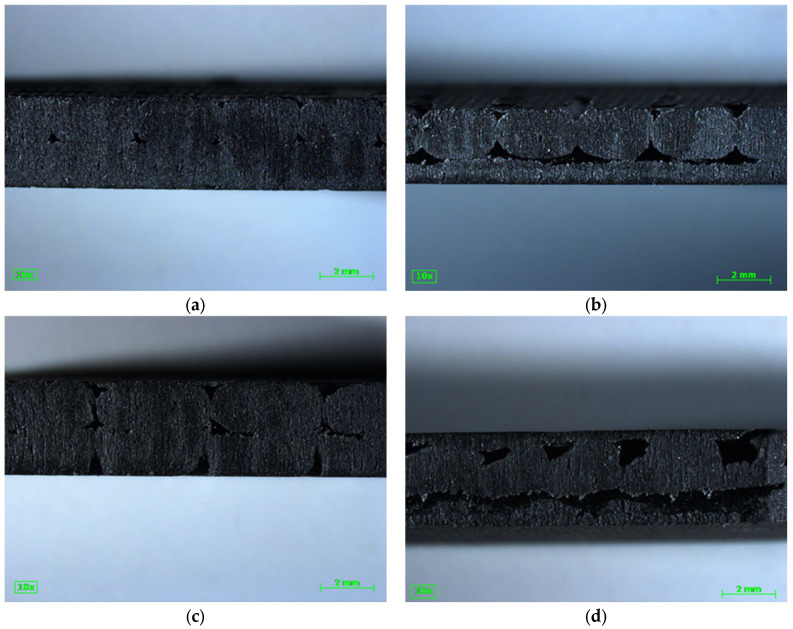
Side view of tested ASTM D638-14 Type V specimens, with scale bar of 2 mm: (**a**) vertical specimen tested at 50 °C with one 0° layer (top with round beads visible) and one 90° layer (bottom with solid bead); (**b**) horizontal specimen tested at 50 °C with one 0° layer (top with round beads visible) and one 90° layer (bottom with solid bead); (**c**) diagonal specimen tested at 50 °C showing a 45° and −45° layer, with both layers showing slightly wider elliptical beads due to cutting at angle; (**d**) vertical specimen tested at room temperature with one 0° layer (middle, showing roughly rounded beads) sandwiched between two 90° layers (straight section along top and bottom).

**Figure A2:**
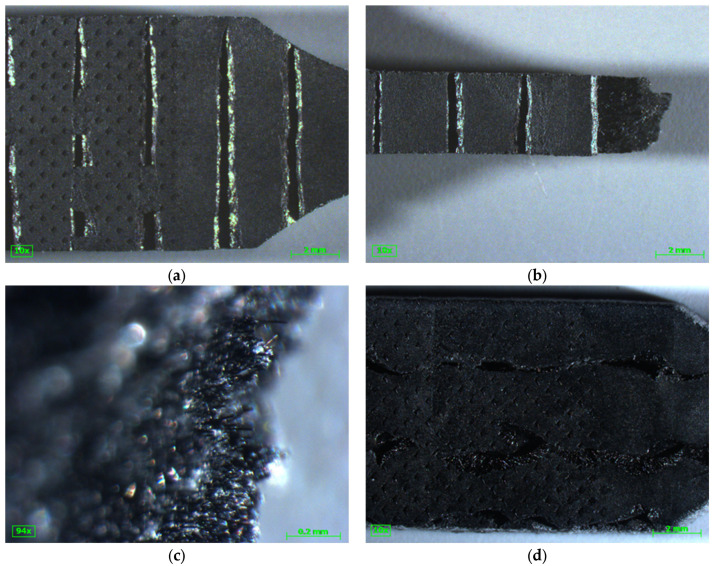
ASTM D638-14 Type V specimen horizontal tested at 50 °C: (**a**) 0° layer showing slight 90° layer at ×10 magnification, scale bar of 2 mm. Note that grid indentations on left half are a result of tensile testing grip; (**b**) failure with 0° layer on top, with 0° failing at curved layer and jagged failure of 90° layer at ×10 magnification, scale bar of 2 mm; (**c**) failure at 90° layer showing carbon fiber, scale bar of 0.2 mm; (**d**) 90° layer, scale bar of 2mm. Note that grid indentations on left half are a result of tensile testing grip.

Figure 13 shows the side cross-sectional views of the specimen, with the view perpendicular to the 90° axis, as shown in Figure 12. The x-direction is along the length of the picture, the z-direction is along the height, and the y-direction is into the page. Most diagonal specimens contained approximately one full layer at 45° and another full layer perpendicular at −45° (Figure 13c), while most horizontal specimens contained approximately one full layer of 0° and 90° (Figure 13b). Specimens did not generally contain exactly one layer each; for example, a horizontal specimen would have a 0° layer, then a 90° layer, and then an incredibly thin section of another 0°, leaving a faint imprint (Appendix C Figure A2a). Vertical specimens were slightly different. Some had the same pattern: a 90° layer, a 0° layer, and then an incredibly thin 90° layer (Figure 13a); other specimens had a 0° layer sandwiched between two half 90° layers (Figure 13d). The void space size and spatial distribution varied by (1) direction (vertical (Figure 13a), horizontal (Figure 13b), and diagonal (Figure 13c)) and (2) by sample (Figure 13a vs. Figure 13d), and the (3) location sample was taken from the panel. Part of the difference in direction was due to the water jet exposing the void space at different angles. Figure 13c shows longer elliptical beads for 45° and −45° layers due to being cut at a 45° angle, while Figure 13a,b show shorter elliptical beads for their 0° layer due to being cut at a 0° angle perpendicular to the bead. Some of the variability is likely caused by differences in heating and cooling during the printing process (i.e., the localized effect of the thermal history of a print on the void space) for both specimen of the same type and different types. Figure 13a shows consistent elliptical beads (0° layer) bonded to a smooth bead (90° layer) with small void spaces while Figure 13d shows uneven elliptical beads (0° layer) bonded to a thin single bead portion (upper 90° layer) and partially bonded to a thicker section (lower 90° layer) with large rough void space. The difference in decking created the difference in bead thickness for a given section. Additionally, the slight unevenness of the print bed may have affected the compaction, resulting in some void space variability across the panel. While the difference in the direction of the cut is expected to result in a difference in the void space displayed at the cross-section, the difference between the two vertical specimen indicates that there may be significant variance in the void space between specimens. This difference could be caused by using samples from different panels or from the void space variation within the print itself (likely caused by the thermal history and compaction varying by location).

**Figure 14:**
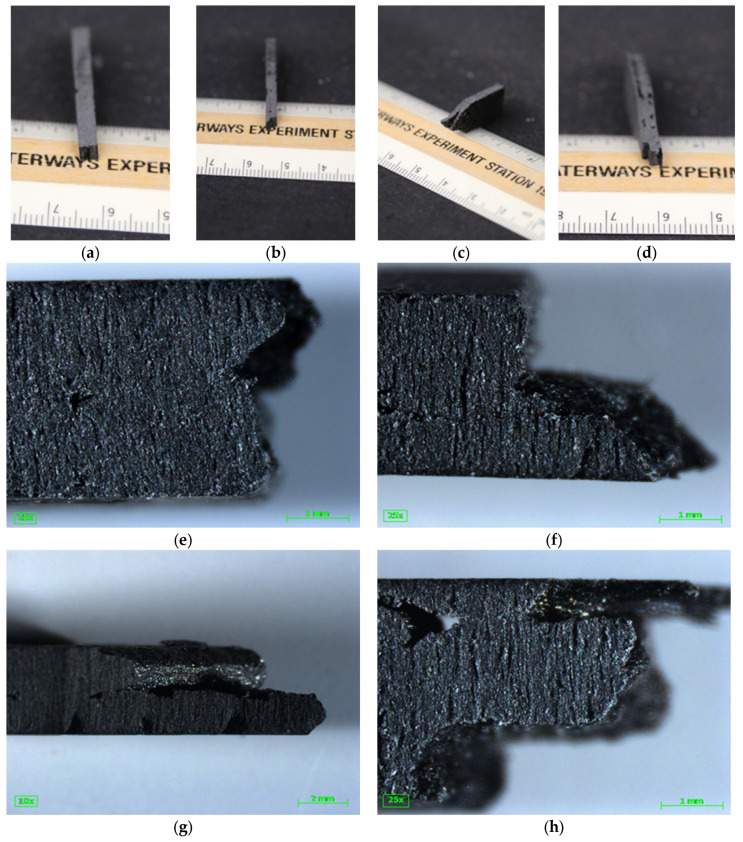
Tested ASTM D638-14 Type V specimens: (**a**) vertical specimen tested at 50 °C with one 90° layer and one 0° layer showing relatively clean failure along 0° (interlayer adhesion zone) and jagged rough failure along 90° (failure of printed bead), scale in cm; (**b**) horizontal specimen tested at 50 °C showing relatively clean failure along 0° and rough jagged failure along 90°, scale in cm; (**c**) diagonal specimen tested at 50 °C showing clean failures along the 45° and −45° layer lines, scale in cm; (**d**) vertical specimen tested at room temperature with one 0° layer sandwiched between two 90° layers, showing relatively clean failure at the 0° layer and two jagged failures on the outside 90° layers, scale in cm; (**e**) 14a magnified with 90° on top and 0° on bottom, scale bar of 1 mm; (**f**) 14b magnified with 0° on top and 90° on bottom, scale bar of 1 mm; (**g**) 14c magnified with −45° and 45° layers, scale bar of 2 mm; (**h**) 14d with magnified with thin 90° on top, 0° layer in the middle and half 90° layer on the bottom, scale bar of 1 mm.

The failure pattern of the tensile specimens, shown in Figure 14, provides some explanation of the difference in tensile strength. There was some commonality in the failure patterns. First, 0° layers broke relatively cleanly along the interlayer adhesion zone between layers (Figure 14a,b,d,e,f,h), where the applied force was perpendicular to the direction of strength. The 90° direction, where the applied force was parallel to the printed bead, showed a jagged rough failure where the bead itself pulled apart (Figure 14a,b,d). Clean failures along the interlayer adhesion zone occurred at both the 45° and −45° layers (Figure 14c,g). While the vertical (Figure 14a,d,e,h) and horizontal (Figure 14b,f) specimens exhibited both clean failures and jagged failures, the diagonal (Figure 14c,g) specimens only exhibited clean failures along the bead adhesion zone. Interlayer adhesion is known to be weaker in tension than the direction of printing for FGF LFAM, though the interlayer strength is commonly reported in the z-direction [15,16,17,40] and the Figure 14 shows failures in the x-y plane. Sánchez et al., 2020 reported that specimens printed in the X direction failed through the bead, while specimens in the Z direction failed along the interlayer adhesion zone [40], with the X direction comparable to the 90° direction and the Z direction comparable to the 0° direction. Additionally, Sánchez et al., 2020 reported that carbon fiber limited bonding between the layers for LFAM ASA CF20% [40], and Pintos et al., 2024 reported that the addition of carbon fiber decreased the interlayer adhesion strength for LFAM ASA [17]. Note that Figure 14a–c,e–g shows the failure of two full printed beads, while Figure 14d,h shows the failure of one full bead and two half beads, where the full bead was in the middle and the half beads were on the outside.

For the diagonal specimens, both failures occurred along the weaker portion of the print (interlayer adhesion zone) while, for the horizontal and vertical, only one failure occurred along the weaker section and the second failure occurred along the stronger section. It follows that the diagonal specimens, being reliant mainly on interlayer adhesion strength, would have a lower tensile strength than the vertical and horizontal specimens, which relied on both material strength and interlayer adhesion strength.

While the difference between the vertical and horizontal specimens is not fully understood, it is proposed that the higher ultimate tensile strength in the vertical specimens could be the result of some 90° half bead specimens resulting in better strength and demonstrating a cascading failure that allowed two separate portions (interlayer strength and one of the half beads) to fail before complete failure. Additionally, there was likely some variation in the void space that negatively affected the horizontal specimens. While a 0°/90° print orientation results in two opposing layers, the layers are affected by the printing process. For example, the distance from the print bed is known to affect the void space [18]. The horizontal samples may have had more or larger void space in their 0° direction (same printed bead as the 90° direction in vertical specimens) than vertical samples in the 0° direction (same printed bead as the 90° direction in horizontal specimens) as a result of their location on the z-axis. The full confirmation of either theory is a potential research need.

For Young’s modulus at room temperature, vertical specimens (5.35 GPa) are on average ~1.5 GPa greater than both the horizontal (3.72 GPa) and diagonal (3.67 GPa) specimens, which have a similar Young’s modulus. Given that the horizontal specimens have a greater average tensile strength than diagonal specimens, it would be expected that the horizontal would have a greater Young’s modulus; however, this is not the case. Young’s modulus is the ratio of stress to strain, and is the slope of a material’s elastic region [13]. It follows that for horizontal specimens to have a similar Young’s modulus to diagonal specimens, given the higher ultimate tensile strength seen in horizontal samples, that horizontal specimen needs a strain that is proportionally higher than the diagonal strain to achieve the same Young’s modulus. The difference in strain is explained by the meso-structure. The diagonal samples exhibit less strain because failure occurs along the weak interlaminar zone for both layers; meanwhile, horizontal specimens contain a 0° layer, which will fail due to interlaminar weakness and a 90° layer, which exhibits more ductility before failure. The horizontal and vertical specimens have similar average strains due to a similar meso-structure, but because the vertical specimens have higher strength, the vertical specimens also have a higher average Young’s modulus. For horizontal specimen, the weaker strength at the same strain results in a lower Young’s modulus than the vertical samples, and a similar ratio of strength to strain results in a similarity to the diagonal samples’ Young’s modulus, despite dissimilar strength. Finally, note that above T_g_, the strain increases for all the samples, leading to an overall decrease in Young’s modulus. See Appendix D for average strain values.

The difference in the room temperature averages of the specimens taken from the x-y plane in the diagonal, horizontal, and vertical direction can be explained by the LFAM meso-structure.

### 3.5. Stress Concentration Calculation

Another way to view the difference in tensile strength is through the difference between the maximum stress felt at the tip of the stress concentration for diagonal, horizontal, and vertical specimens. The elliptical assumption and Equation (4) below were adapted from Callister and Rethwisch [13]. First, for simplicity, it was assumed that a single void space between two printed beads in the specimen layer and the beads printed in the next layer can be approximated as an elliptical hole. The plane of reference was the thin long side of the tensile specimen where a cross-section of the void space was visible (see Figure 13). The progression of the crack propagation along the length of the void space (into page of Figure 13) was not considered. It was also assumed that stress was applied by a universal testing machine along the 90° axis of the tensile specimen (Figure 12) and was called $\sigma_{t}$. Equation (4) approximates the maximum stress along a crack tip and has been adapted from Callister and Rethwisch [13] to fit a FFF/FGF problem.

(4) $$\sigma_{v}=2\sigma_{a}\sqrt{\frac{w}{c}}$$

where

$\sigma_{v}$ = maximum stress at the edge of a void space;

w = half the length of the void space in the plane of interest;

c = curvature radius of the void space crack tip;

$\sigma_{a}$ = stress applied perpendicular to the void space.

Three conditions of the void space were considered: the void space parallel to 90°, the void space parallel to 45°, and the void space parallel to 0°. It was assumed that the −45° was equivalent to the 45° condition in all the cases. (Refer to Figure 13 for the direction of the void space on tensile specimen.) For the simplicity of mathematical calculation, the void space for 0°, 45°, and 90° can be assumed to have the same curvature (d) given that all specimens were taken from the same panels with the same print parameters, with only the direction of removal varying. Furthermore, it was assumed that all the cross-sectional void space, viewed from the side of the tensile specimen (Figure 13 for perspective), has the same length, for which half was referred to as a. While not a perfect assumption, given the same print parameters and panels, it serves as a reasonable approximation. Given that $\sigma_{t}$ is applied parallel to the void space in the 90° direction

(5) $$\sigma_{a,90{}^{\circ}}=0,$$

so

(6) $$\sigma_{v,90{}^{\circ}}=0.$$

For the void space in the 0° direction,

(7) $$\sigma_{a,0{}^{\circ}}=\sigma_{t}$$

because the stress was directly perpendicular to the void space. For the void space in the 45°,

(8) $$\sigma_{a,45{}^{\circ}}=\frac{\sigma_{t}}{\sqrt{2}\;}.$$

Assuming one void space between layers, then for 0°,

(9) $$\sigma_{v,0{}^{\circ}}=2\sigma_{t}\sqrt{\frac{a}{d}}$$

and for 45°,

(10) $$\sigma_{v,45{}^{\circ}}=\frac{2}{\sqrt{2}}\sigma_{t}\sqrt{\frac{a}{d}}.$$

The applied force at the crack tip was greater for the 0° direction than the 45° direction. In general, considering the stress applied to one crack tip of one void space,

(11) $$\sigma_{v,0{}^{\circ}}>\sigma_{v,45{}^{\circ}}>\sigma_{v,90{}^{\circ}}.$$

The next consideration was a two-layered test specimen containing two external surface cracks (resulting from the decking and exposure of the printed void space) and one internal void space shared by the layers. One external crack and half of the internal void space were assigned to one layer each. The test specimens existed in two rough variations: one 0° layer and one 90° layer (vertical and horizontal) and then one 45° layer and one −45° layer (diagonal). For simplicity, the total stress of a single location was combined into one term called $\sigma_{v,combined}$ to demonstrate the effect of the void space direction. The full failure of a specimen was assumed to occur after both layers were broken, and $\sigma_{v,combined}$ was used as an equivalence term, where a higher value results in earlier failure. For 0°,90° specimens,

(12) $$2\sigma_{v,0{}^{\circ}}+2\sigma_{v,90{}^{\circ}}=4\sigma_{t}\sqrt{\frac{a}{d}}$$

while for 45°, −45°,

(13) $$2\sigma_{v,45{}^{\circ}}+2\sigma_{v,45{}^{\circ}}=\frac{8}{\sqrt{2}}\sigma_{t}\sqrt{\frac{a}{d}}.$$

In general, the combined stress at a single section containing two external surface cracks and one internal void space,

(14) $$\sigma_{v,combined,\;diagonal}>\sigma_{v,combined,horizontal}=\sigma_{v,combined,\;vertical},$$

demonstrating that the cracking stress felt by the diagonal specimens was greater than the horizontal and vertical specimens.

This analysis leads to the following conclusions. First, vertical specimens with two half layers 90° and one full layer 0° would have two internal void spaces and no external cracks, and failure would need to occur across three layers rather than two. Second, the variation in void space would lead to variation in the shape and size of stress concentrations, leading to local variation within the test specimen. While the elliptical void space was assumed for simplicity, as can be seen in Figure 13, the void space was not elliptical and a more accurate calculation could be made by assuming a more accurate shape that includes the void space sharpness. Third, the variation of the void space size, the void space in 3D space, and the process of failure, while important, were not directly considered in the math outlined above. Finally, $\sigma_{v,combined}$ was an equivalence term used to help explain the weakness of diagonal specimens when compared to vertical and horizontal specimens.

### 3.6. The Difference between Injection-Molded Strength and FGF LFAM Print Strength

Both Pintos et al., 2024 and Pintos, León, and Molina 2024 report achieving equivalent or higher tensile strength for specimens printed with LFAM when compared to injection molding [15,17]. Injection-molded specimens generally have higher ultimate tensile strengths than desktop 3D-printed specimens [18,35] and are reported to have greater ultimate tensile strengths than FGF LFAM in Sánchez et al., 2020 [40] and in this paper. A number of causes might explain the differences. Specimens in this work were decked to 4 mm, while Pintos et al., 2024, Pintos, León, and Molina 2024, and Sánchez et al., 2020 did not report machining to achieve desired specimen thickness [15,17,40]. The decking likely opened stress concentrations already extant in the specimens [25], potentially explaining part of the difference seen for this work, but likely not the difference seen in Sánchez et al., 2020 [40]. Both Pintos, León, and Molina and Pintos et al., 2024 printed with a layer height of 1 mm [15,17], which likely resulted in smaller voids than printing at a 2 mm layer height in this work or the 2.5 mm layer height seen in Sánchez et al., 2020 [40]. Theoretical stress-concentration factors are based on geometry and can be applied in static loading to brittle materials (strain at failure < 0.05) [44]. Because PETG CF30% FGF LFAM exhibits evidence of having strain at failure less than 0.05 [25], a stress concentration related to the ratio of the void space to bead diameter or the void space to the full part could also explain the difference seen. Additionally, the current work optimized for a smooth round bead, which may have resulted in interlayer weakness, due to a higher void space to part ratio. Note that high extrusion multiplier results in increased interlayer strength for LFAM [16].

There was also a difference in the test specimen type. Pintos et al., 2024 used a 1BA specimen [17], Pintos, León, and Molina 2024 used test specimens from ISO 921 [15], Sánchez et al., 2020 used UNE-116005:2012 [40], and the current work used ASTM D638-14 Type V specimens. Also, the infill direction of the XY plane and the direction of specimen removal would affect the reported strength. Pintos, León, and Molina 2024 appear to have printed in the 0° only and removed specimens along the direction of the printed bead [15]. Pintos et al., 2024 appear to have printed in the 0° direction and do not report the direction of specimen removal [17], though, given the high ultimate tensile strengths reported, specimens were likely removed along the direction of the printed bead. For Sánchez et al., 2020, the print infill and specimen removal direction are not specified [40]; however, it is assumed, given the discussion on the printed bead serving as the direction of strength and the figures provided, that the infill was 0° and there specimen was removed in the direction of the printed bead. Testing with multiple layers parallel to the applied force, as done in Pintos, León, and Molina 2024 and Pintos et al., 2024 [15,17], would result in a higher strength than testing with one layer parallel to applied force and one perpendicular to applied force (where stress concentrations become more significant and weaken the part) as done in the current work; however, that does not explain the difference seen in Sánchez et al., 2020 [40], in which specimen are also assumed to test in the direction of strength.

Next, the difference in material choice is considered. While Pintos et al., 2024 (ASA and ASA CF23) [15] and Pintos, León, and Molina 2024 (PETG and PET) [17] have equivalent or higher strength than injection-molded samples in some cases, Sánchez et al., 2020 (ASA and ASA CF20) [40] and this work (PETG CF30%) show lower strength than injection-molded samples; however, there is some nuance to these findings. In Pintos et al., 2024 [17], ASA CF23 with shorter fibers and ASA exhibited a similar strength for injection molding and the FGF LFAM, while ASA CF23 with longer fibers exhibited higher ultimate tensile strength by ~20 MPa for injection molding when compared to the FGF LFAM. Additionally, the injection-molded ASA CF23 with shorter fibers exhibited a lower strength than the injection-molded ASA CF23 with longer fibers by ~31 MPa. Fiber is known to orient in injection-molded fiber/composites [27], indicating that the material choice and orientation of the fiber affects both the injection molding and the LFAM ultimate tensile strength. Meanwhile, in Pintos, León, and Molina 2024, injection-molded tensile strength and LFAM tensile strength in the XY plane for PET2 and PET3 are within ~7 MPa, while PETG is within 1 MPa. In Sánchez et al., 2020, while the injection-molded ASA CF20 shows ~15 MPa higher tensile strength than the specimen printed in the X direction, injection-molded ASA only exhibits a difference of ~5 MPa [40], indicating that the carbon fiber affects the difference between injection molding and FGF LFAM. The current work exhibits, on average, a difference of ~85 MPa in the best case and ~110 MPa in the worst case, which is larger than ranges previously presented in the literature. As discussed above, the difference in strength seen in this work when compared to the literature is likely due to decking opening the void space, print parameter optimization choice, test direction, and infill direction, as well as material choice (size and wt.% of carbon fiber), which potentially increased the disparity between injection molding and FGF LFAM. Similarity, between injection-molded and FGF LFAM ultimate tensile strength depends partially on material properties.

The results above indicate a couple observations. First, injection-molded samples do not always have a higher ultimate tensile strength than FGF LFAM specimen. Second, understanding fiber material properties (length, length distribution), composite properties (wt.%, fiber distribution), and processing effects (fiber orientation, fiber distribution) for both injection molding and FGF LFAM will allow for better understanding of the discrepancies and similarities seen between the ultimate tensile strength. Finally, further study is needed to understand the effect of the layer height and the resulting void space on the ultimate tensile strength. The difference in meso-structure design, testing choice, and material choice likely resulted in the dissimilar strengths of injection-molded specimens and specimens printed in the x-y plane between Pintos et al. and Pintos, León, and Molina 2024, Sánchez et al., 2020, and the current work [15,17,40].

## 4. Conclusions

Statistical analyses of Young’s modulus and ultimate tensile strength results for PETG CF30% tensile specimens produced by four manufacturing processes (injection molding and LFAM panel, taken in the horizontal, diagonal, and vertical directions) and tested at six temperatures (RT, 40 °C, 50 °C, 60 °C, 70 °C, and 80 °C) were completed.

- For the tensile data, the variance observed within each specimen set did not follow any consistent trend regarding testing temperature. Diagonal specimens generally had high coefficients of variation and injection-molded specimens generally had low coefficients of variation when compared to other manufacture methods. Increasing temperature resulted in a decrease in the ultimate tensile strength, though injection-molded specimens and diagonal specimens were statistically distinct from horizontal and vertical specimens at 80 °C.
- For Young’s modulus, there was inconsistent variance within each specimen set. All manufacture methods showed low standard deviations at 80 °C, especially the LFAM specimens. Both diagonal and injection-molded specimens had high coefficients of variation at 80 °C. Young’s modulus became statistically similar for all manufacturing methods at 80 °C.
- Both the manufacturing process and the testing temperature statistically influenced the measured Young’s modulus and ultimate tensile strength when the data set was analyzed holistically. The effect of the manufacturing process on the ultimate tensile strength was 44.2% and the effect of temperature was 47.4%, indicating that both had a strong effect on the tensile strength. For Young’s modulus, the effect of the manufacturing process was 13.9% while the effect of the temperature was 82.6%, indicating that the temperature was highly significant, likely due to the effect of the transition from glassy to rubbery above the glass transition temperature.
- The difference in room temperature strength between diagonal, horizontal, and vertical specimens was a result of the 3D-printed meso-structure. The diagonal specimens broke cleanly for both layers along the layer lines, indicating that the lower strength was caused by relying on interlayer strength. The vertical and horizontal specimens both relied on material strength and interlayer strength. While not fully understood, the vertical strength could be due to some specimens containing two half layers and one full layer, requiring more energy to fail, or due to the variation in the void space.
- The maximum stress at the crack tip was calculated for the diagonal, horizontal, and vertical specimens. The diagonal specimens had the highest equivalent combined stress, indicating that the diagonal specimens would have the weakest tensile strength.
- Both the material properties and meso-structural design must be carefully considered when designing for LFAM.

While the LFAM followed expected trends in manufacturing strength (injection molding was stronger than 3D printing) and in thermomechanical behavior (above T_g_, there was a decrease in Young’s modulus and strength), there was some nuance caused by the meso-structure (void space variation and testing direction). Further research should consider the effect of print parameters on void space variation, the void space variation between specimens and across a single large print, and the effect of void space variation on variation in tensile strength. 

## Figures and Tables

**Table 1:** Print parameters for panels. Adapted from Martin et al., 2024 [25] with permission of lead author.

Print Parameter	Panel I
Material	PETG CF30%
Drying State	Undried, Dried
Nozzle Diameter (on printer) (mm)	3
Nozzle Temperature (°C) (PID)	250
Print Bed Temperature (°C) (PID)	80
M3 Motor (Mach4) (counts/unit)	276
Layer Height (mm)	2
Initial Layer Height (mm)	2
Maximum Volumetric Extrusion (mm^3^/s)	500
Maximum Print Speed (mm/s)	500
Infill Print Speed (mm/s)	600
(In)Fill Pattern	Rectilinear
(In)Fill Angle	0/90
Infill Percentage (%)	100
Infill–Perimeter Overlap (%)	20
Filament Diameter (mm)	5.5
Nozzle Diameter (mm)	3
Extrusion Multiplier	1
Perimeter (beads)	3
Agitator	Dried: 3 off 1 on ^1^

^1^ Refers to ratio of time that agitator is off (3) vs. on (1).

**Table 2:** Average ultimate tensile strength, standard deviation, coefficient of variation, and specimens tested for all testing temperatures and manufacture methods. Average ultimate tensile strength data previously presented graphically in Martin et al., 2024 [25].

Direction/Specimen Type	Temperature (°C)	Average Ultimate Tensile Strength (MPa)	Standard Deviation	Coefficient of Variation (%)	Number of Specimens Tested ^1^
Injection-Molded	RT	123.39	4.26	3.45	5
	40	95.36	5.87	6.15	5
	50	95.16	11.04	11.61	5
	60	51.96	2.88	5.54	5
	70	38.30	5.59	14.61	5
	80	9.89	2.15	21.78	4
Horizontal	RT	22.89	6.10	26.67	4
	40	23.95	5.36	22.38	5
	50	26.33	1.68	6.38	4
	60	24.47	9.26	37.85	4
	70	15.08	1.44	9.54	4
	80	5.31	0.26	4.85	4
Vertical	RT	37.98	0.95	2.50	4
	40	30.89	6.74	21.82	5
	50	33.72	5.99	17.77	4
	60	30.43	4.40	14.46	4
	70	12.04	0.54	4.52	4
	80	4.54	0.37	8.20	4
Diagonal	RT	14.17	3.46	24.41	4
	40	19.04	6.00	31.51	5
	50	15.31	4.43	28.97	4
	60	18.50	2.45	13.26	4
	70	7.07	0.74	10.44	4
	80	2.45	0.61	24.90	4

^1^ Number of specimens, as per Martin et al. [25].

**Table 3:** Average Young’s modulus, standard deviation, coefficient of variation, and specimens tested for all testing temperatures and manufacture methods. Average ultimate tensile strength data previously presented graphically in Martin et al., 2024 [25].

Direction/Specimen Type	Temperature (°C)	Average Young’s Modulus (GPa)	Std. Dev.	Coefficient of Variation (%)	Number of Specimens Tested ^1^
Injection-Molded	RT	8.56	0.24	2.83	5
	40	8.00	0.65	8.12	5
	50	9.42	0.45	4.75	5
	60	6.85	0.79	11.53	5
	70	6.05	0.95	15.74	5
	80	0.10	0.13	125.33	4
Horizontal	RT	3.72	0.15	4.00	4
	40	3.52	0.24	6.82	5
	50	3.92	0.23	5.94	4
	60	4.13	1.20	29.17	4
	70	3.16	0.37	11.82	4
	80	0.03	0.0016	6.18	4
Vertical	RT	5.35	0.52	9.78	4
	40	5.02	0.53	10.60	5
	50	4.84	0.76	15.61	4
	60	4.58	0.43	9.40	4
	70	2.37	0.20	8.51	4
	80	0.02	0.0020	8.39	4
Diagonal	RT	3.67	0.19	5.26	4
	40	3.52	0.08	2.39	5
	50	3.14	0.44	14.07	4
	60	3.11	0.15	4.91	4
	70	1.89	0.11	6.06	4
	80	0.03	0.030	101.91	4

^1^ Number of specimens, as per Martin et al. [25].

**Table 4:** Two-way ANOVA for ultimate tensile strength.

Source	SS ^1^	DF ^2^	MS ^3^	F ^4^	P ^5^
Manufacture	17,100	3	5690	440	0.000
Temperature	18,300	5	3660	283	0.000
Interaction	2320	15	154	11.9	0.000
Error	932	72	12.9		
Total	38,600	95			

^1^ Sums of squares; ^2^ degrees of freedom; ^3^ mean squares = SS/DF; ^4^ test statistic = SS(treatment)/SS(error); ^5^ probability of null hypothesis that all means are equal is true. NOTE: Data was transformed using λ = 0.231 to improve normality and homogeneity of variance.

**Table 5:** One-way ANOVAs for statistical differences of tensile strength between testing temperatures blocked by manufacturing process.

Diagonal (λ = 0.107)					
Source	SS	DF	MS	F	P
Temperature	1140	5	228	42.8	0.000
Error	95.8	18	5.32		
Total	1240	23			
Horizontal (λ = −1.05)					
Source	SS	DF	MS	F	P
Temperature	6290	5	1260	135	0.000
Error	168	18	9.32		
Total	6460	23			
Vertical (λ = −0.238)					
Source	SS	DF	MS	F	P
Temperature	7330	5	1470	305	0.000
Error	86.5	18	4.81		
Total	7420	23			
Injection-Molded (λ = 0.428)					
Source	SS	DF	MS	F	P
Temperature	37,700	5	7540	239	0.000
Error	568	18	31.6		
Total	38,300	23			

**Table 6:** Statistical grouping of manufacture method when compared to temperature for injection-molded and 3D-printed specimens for ultimate tensile strength.

Testing Temperature (°C)	Group A	Group B	Group C
RT	IM (123 MPa)	V (38.0 MPa)	D/H (18.6 MPa)
40	IM (96.7 MPa)	H/V (28.9 MPa)	D/H (20.5 MPa)
50	IM (95.6 MPa)	H/V (30.0 MPa)	D/H (20.8 MPa)
60	IM (52.7 MPa)	D/H/V (24.5 MPa)	
70	IM (37.1 MPa)	H/V (13.6 MPa)	D (7.07 MPa)
80	IM (9.89 MPa)	H/V (4.93 MPa)	D (2.44 MPa)

**Table 7:** Two-way ANOVA for Young’s modulus.

Source	SS	DF	MS	F	P
Manufacturing	77.9	3	26.0	305	0.000
Temperature	464	5	92.8	1090	0.000
Interaction	13.5	15	0.903	10.6	0.000
Error	6.14	72	0.0852		
Total	562	95			

NOTE: Data transformed using λ = 0.400 to improve normality and homogeneity of variance.

**Table 8:** One-way ANOVA for statistical differences of Young’s modulus between testing temperatures blocked by manufacturing process.

Diagonal (λ = 0.513)					
Source	SS	DF	MS	F	P
Temperature	46.7	5	9.34	369	0.000
Error	0.456	18	0.0253		
Total	47.2	23			
Horizontal (λ = −0.176)					
Source	SS	DF	MS	F	P
Temperature	393	5	78.7	2020	0.000
Error	0.700	18	0.0389		
Total	394	23			
Vertical (λ = −0.0386)					
Source	SS	DF	MS	F	P
Temperature	342	5	68.3	2180	0.000
Error	0.563	18	0.0313		
Total	342	23			
Injection-Molded (λ = 0.618)					
Source	SS	DF	MS	F	P
Temperature	280	5	56.0	281	0.000
Error	3.58	18	0.199		
Total	283	23			

**Table 9:** Statistical grouping of manufacture method when compared to temperature for injection-molded and 3D-printed specimens for Young’s modulus.

Testing Temperature (°C)	Group A	Group B	Group C
RT	IM (8.56 GPa)	V (5.35 GPa)	D/H (3.70 GPa)
40	IM (8.21 GPa)	V (5.26 GPa)	D/H (3.50 GPa)
50	IM (9.56 GPa)	H/V (4.38 GPa)	D/H (3.53 GPa)
60	IM (7.13 GPa)	D/H/V (3.94 GPa)	
70	IM (6.28 GPa)	H (3.16 GPa)	D/V (2.13 GPa)
80	IM/D/H/V (0.0515 GPa)		

**Figure A1:**
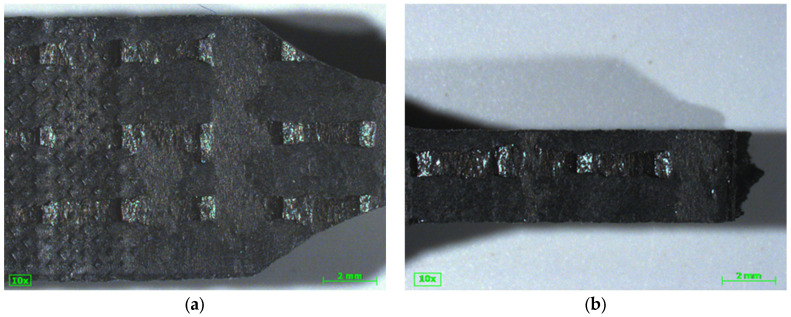

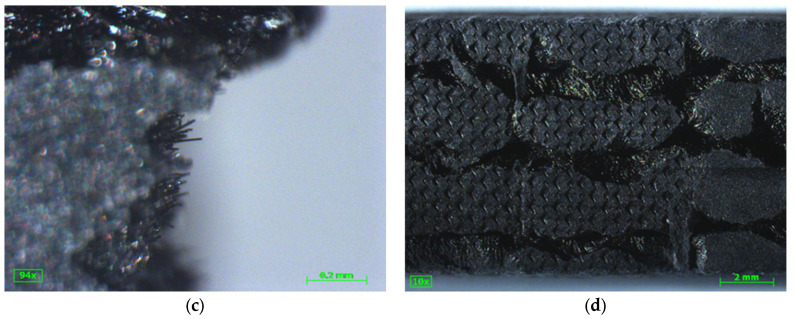
ASTM D638-14 Type V specimen vertical tested at 50 °C: (**a**) 0° layer showing slight 90° layer at ×10 magnification, scale bar of 2 mm. Note that grid indentations on left half are a result of tensile testing grip; (**b**) failure with 0° layer on top, with 0° failing at curved layer and jagged failure of 90° layer at ×10 magnification, scale bar of 2 mm; (**c**) failure at 90° layer showing carbon fiber, scale bar of 0.2 mm; (**d**) 90° layer showing slight 0° layer at ×10 magnification, scale bar of 2 mm. Note that grid indentations on left half are a result of tensile testing grip.

**Figure A3:**
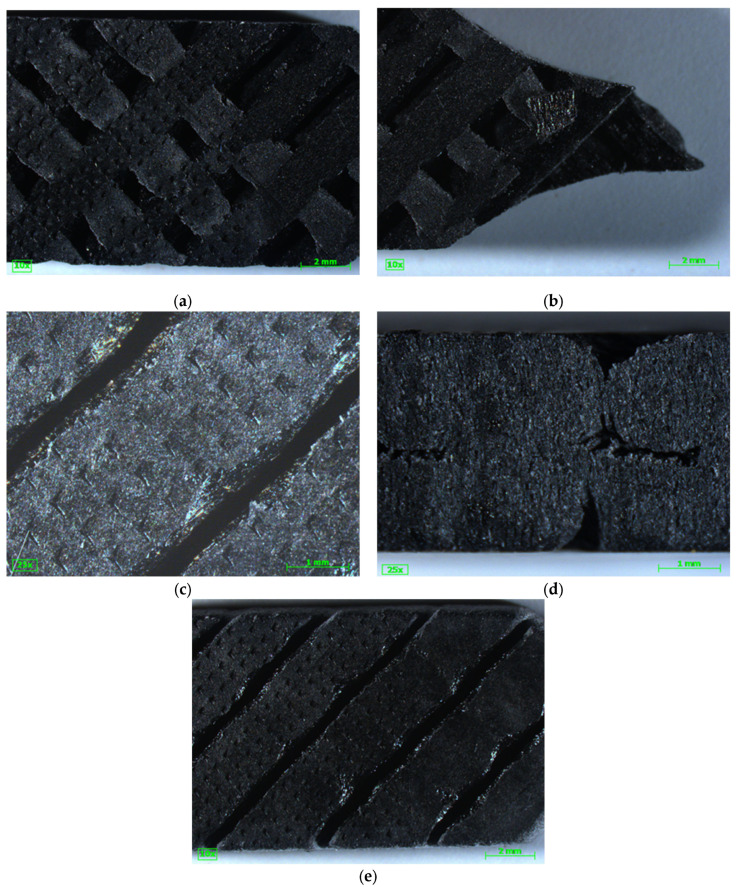
ASTM D638-14 Type V specimen diagonal tested at 50 °C: (**a**) −45° layer with very thin 45° layer, scale bar of 2 mm. Note that grid indentations on left half are a result of tensile testing grip; (**b**) smooth failure at −45° on top and 45° on bottom, scale bar of 2 mm; (**c**) −45° layer lines showing void space, scale bar of 1 mm. Note that grid indentations on printed bead are a result of tensile testing grip; (**d**) close up showing −45° and 45° beads at ×25 magnification, scale bar of 1 mm; (**e**) 45° layer, scale bar of 2 mm. Note that grid indentations on left half are a result of tensile testing grip.

**Figure A4:**
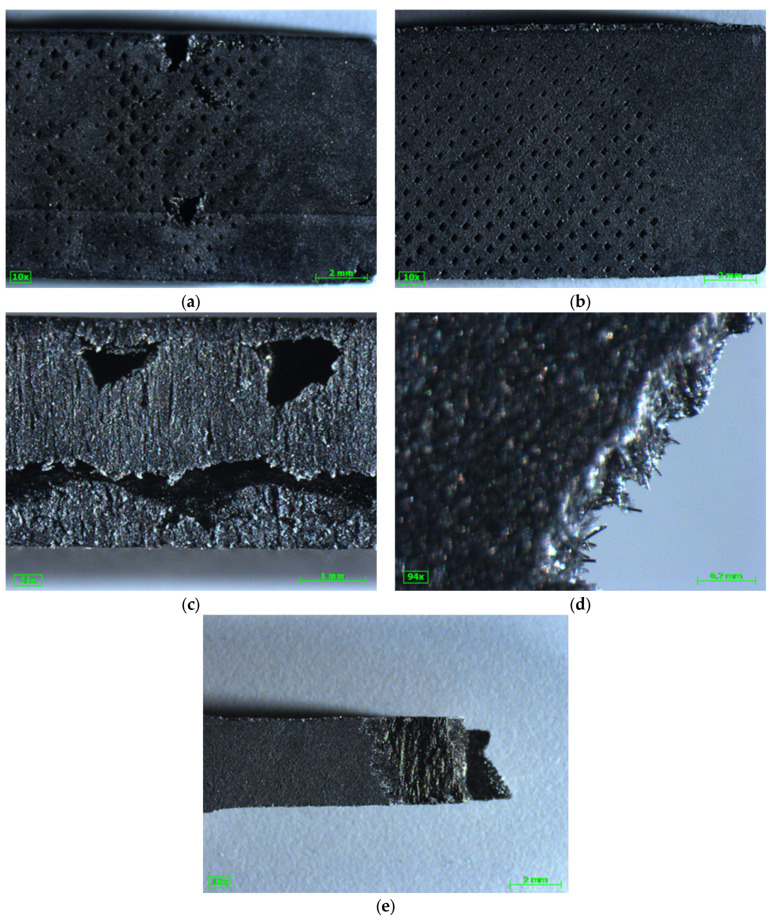
ASTM D638-14 Type V specimen vertical tested at room temperature: (**a**) 90° layer of longer failure, scale bar of 2 mm. Note that grid indentations on left half are a result of tensile testing grip. Additionally note that the right shows smooth material without any visible void space. Two voids are visible in the middle of the picture; (**b**) 90° layer of shorter failure, scale bar of 2 mm. Note that grid indentations on left half are a result of tensile testing grip. Additionally note that the right shows smooth material without any visible void space; (**c**) close-up showing 0° layer sandwiched between two half 90° layers at 25× magnification, scale bar of 1 mm; (**d**) carbon fiber on long 90° failure, scale bar of 0.2 mm; (**e**) short jagged 90°, middle smooth 0° failure, and long 90° failure, scale bar of 2 mm.

## Data Availability

The original contributions presented in the study are included in the article. Further inquiries can be directed to the corresponding author.

## Appendix A. Mean, Tukey Statistic Mean, and Average Difference Tables for Tensile Strength

Table A1 shows the sample means of tensile strength for a set of four specimens for diagonal, horizontal, vertical, and injection molding. Table A2 shows the Tukey test statistic and the absolute difference between the sample means for tensile strength for diagonal, horizontal, vertical, and injection molding. Table A3 shows one-way ANOVA for statistical differences of tensile strength between manufacture methods blocked by the testing temperature. Table A4 shows the Tukey test statistic and the absolute difference between sample means for tensile strength blocked by the testing temperature.

**Table A1:** Sample means of tensile strength for a set of four specimens blocked by manufacture method.

Diagonal						
Temperature (°C)	Room	40	50	60	70	80
Mean UTS (MPa)	14.2	16.8	15.3	18.5	7.07	2.45
Horizontal						
Temperature (°C)	Room	40	50	60	70	80
Mean UTS (MPa)	22.9	24.1	26.3	24.5	15.1	5.31
Vertical						
Temperature (°C)	Room	40	50	60	70	80
Mean UTS (MPa)	38.0	33.7	33.7	30.4	12.0	4.54
Injection-Molded						
Temperature (°C)	Room	40	50	60	70	80
Mean UTS (MPa)	123	96.7	95.6	52.7	37.1	9.89

**Table A2:** Tukey test statistic and absolute difference between sample means for tensile strength diagonal, horizontal, vertical, and injection molding.

Diagonal (λ = 0.107)						
Tukey Test Statistic (MPa)		40 °C	50 °C	60 °C	70 °C	80 °C
6.46	Room	1.81	0.74	2.97	6.82	16.7
	40 °C		1.06	1.17	8.62	18.5
	50 °C			2.23	7.56	17.5
	60 °C				9.79	19.7
	70 °C					9.92
Horizontal (λ = −1.05)						
Tukey Test Statistic (MPa)		40 °C	50 °C	60 °C	70 °C	80 °C
8.55	Room	1.04	2.52	0.57	5.88	43.2
	40 °C		1.48	0.47	6.92	44.3
	50 °C			1.96	8.40	45.7
	60 °C				6.44	43.8
	70 °C					37.3
Vertical (λ = −0.238)						
Tukey Test Statistic (MPa)		40 °C	50 °C	60 °C	70 °C	80 °C
6.14	Room	2.16	2.40	4.15	23.1	48.4
	40 °C		0.239	1.99	21.0	46.3
	50 °C			1.75	20.7	46.0
	60 °C				19.0	44.3
	70 °C					25.3
Injection-Molded (λ = 0.428)						
Tukey Test Statistic (MPa)		40 °C	50 °C	60 °C	70 °C	80 °C
15.7	Room	17.4	18.3	53.8	71.2	117
	40 °C		0.993	36.4	53.9	99.6
	50 °C			35.4	52.9	98.6
	60 °C				17.4	63.2
	70 °C					45.7

**Table A3:** One-way ANOVA for statistical differences of tensile strength between manufacture methods blocked by testing temperature.

Room Temperature					
Source	SS	DF	MS	F	P
Manufacturing	30,000	3	9990	544	0.000
Error	220	12	18.4		
Total	30,200	15			
40 °C					
Source	SS	DF	MS	F	P
Manufacturing	16,000	3	5350	225	0.000
Error	286	12	23.8		
Total	16,300	15			
50 °C (λ = 0.348)					
Source	SS	DF	MS	F	P
Manufacturing	9380	3	3130	93.8	0.000
Error	400	12	33.3		
Total	9780	15			
60 °C					
Source	SS	DF	MS	F	P
Manufacturing	2680	3	894	30.2	0.000
Error	355	12	29.6		
Total	3040	15			
70 °C (λ = −0.275)					
Source	SS	DF	MS	F	P
Manufacturing	1170	3	390	170	0.000
Error	27.5	12	2.29		
Total	1200	15			
80 °C (λ = 0.248)					
Source	SS	DF	MS	F	P
Manufacturing	95.3	3	31.8	41.7	0.000
Error	9.13	12	0.76		
Total	104	15			

**Table A4:** Tukey test statistic and absolute difference between sample means for tensile strength blocked by testing temperature.

Room Temperature				
Tukey Test Statistic (MPa)		Horizontal	Vertical	Injection-Molded
11.8	Diagonal	8.71	23.8	109
	Horizontal		15.1	100
	Vertical			85.0
40 °C				
Tukey Test Statistic (MPa)		Horizontal	Vertical	Injection-Molded
13.4	Diagonal	7.27	16.9	79.9
	Horizontal		9.61	72.6
	Vertical			63.0
50 °C (λ = 0.348)				
Tukey Test Statistic (MPa)		Horizontal	Vertical	Injection-Molded
15.9	Diagonal	15.6	23.3	65.4
	Horizontal		7.68	49.8
	Vertical			42.1
60 °C				
Tukey Test Statistic (MPa)		Horizontal	Vertical	Injection-Molded
15.0	Diagonal	5.97	11.9	34.2
	Horizontal		5.96	28.3
	Vertical			22.3
70° C (λ = −0.275)				
Tukey Test Statistic (MPa)		Horizontal	Vertical	Injection-Molded
4.16	Diagonal	12.3	8.98	23.9
	Horizontal		3.34	11.6
	Vertical			14.9
80 °C (λ = 0.248)				
Tukey Test Statistic (MPa)		Horizontal	Vertical	Injection-Molded
2.40	Diagonal	3.59	2.81	6.85
	Horizontal		0.773	3.26
	Vertical			4.04

## Appendix B. Mean, Tukey Statistic Mean, and Average Difference Tables for Young’s Modulus

Table A5 shows the sample means of Young’s modulus for a set of four specimens for diagonal, horizontal, vertical, and injection molding. Table A6 shows the Tukey test statistic and the absolute difference between the sample means for Young’s modulus for diagonal, horizontal, vertical, and injection molding. Table A7 shows one-way ANOVA for statistical differences of Young’s modulus between the manufacture methods blocked by testing temperature. Table A8 shows Young’s modulus statistic and the absolute difference between the sample means for tensile strength blocked by the testing temperature.

**Table A5:** Sample means of Young’s modulus for a set of four specimens for diagonal, horizontal, vertical, and injection molding.

Diagonal						
Temperature (°C)	Room	40	50	60	70	80
Mean E (GPa)	3.67	3.50	3.14	3.11	1.89	0.0291
Horizontal						
Temperature (°C)	Room	40	50	60	70	80
Mean E (GPa)	3.72	3.49	3.92	4.13	3.16	0.0265
Vertical						
Temperature (°C)	Room	40	50	60	70	80
Mean E (GPa)	5.35	5.26	4.84	4.58	2.37	0.0234
Injection-Molded						
Temperature (°C)	Room	40	50	60	70	80
Mean E (GPa)	8.56	8.21	9.56	7.13	6.28	0.127

**Table A6:** Tukey test statistic and absolute difference between sample means for Young’s modulus for diagonal, horizontal, vertical, and injection molding.

Diagonal (λ = 0.513)						
Tukey Test Statistic (GPa)		40 °C	50 °C	60 °C	70 °C	80 °C
0.446	Room	0.099	0.338	0.347	1.24	3.99
	40 °C		0.238	0.247	1.14	3.89
	50 °C			0.00932	0.905	3.65
	60 °C				0.896	3.64
	70 °C					2.75
Horizontal (λ = −0.176)						
Tukey Test Statistic (GPa)		40 °C	50 °C	60 °C	70 °C	80 °C
0.552	Room	0.0917	0.0717	0.0959	0.235	10.9
	40 °C		0.163	0.188	0.144	10.8
	50 °C			0.0242	0.307	11.0
	60 °C				0.331	11.0
	70 °C					10.7
Vertical (λ = −0.0386)						
Tukey Test Statistic (GPa)		40 °C	50 °C	60 °C	70 °C	80 °C
0.495	Room	0.0212	0.182	0.265	1.43	10.4
	40 °C		0.160	0.244	1.41	10.4
	50 °C			0.0832	1.24	10.2
	60 °C				1.16	10.2
	70 °C					8.99
Injection-Molded (λ = 0.618)						
Tukey Test Statistic (GPa)		40 °C	50 °C	60 °C	70 °C	80 °C
1.25	Room	0.265	0.706	1.08	1.77	9.40
	40 °C		0.972	0.816	1.51	9.13
	50 °C			1.79	2.48	10.1
	60 °C				0.692	8.32
	70 °C					7.62

**Table A7:** One-way ANOVA for statistical differences of Young’s modulus between manufacture methods blocked by testing temperature.

Room Temperature					
Source	SS	DF	MS	F	P
Manufacturing	63.3	3	21.1	205	0.000
Error	1.23	12	0.103		
Total	64.6	15			
40 °C					
Source	SS	DF	MS	F	P
Manufacturing	59.3	3	19.8	223	0.000
Error	1.06	12	0.0886		
Total	60.4	15			
50 °C					
Source	SS	DF	MS	F	P
Manufacturing	99.6	3	33.2	139	0.000
Error	2.86	12	0.239		
Total	102	15			
60 °C					
Source	SS	DF	MS	F	P
Manufacturing	35.1	3	11.7	24.1	0.000
Error	5.82	12	0.485		
Total	40.9	15			
70 °C (λ = −0.620)					
Source	SS	DF	MS	F	P
Manufacturing	26.5	3	8.82	91.7	0.000
Error	1.15	12	0.0962		
Total	27.6	15			
80 °C (λ = −0.603)					
Source	SS	DF	MS	F	P
Manufacturing	0.00440	3	0.00147	5.09	0.0168
Error	0.00346	12	0.000288		
Total	0.00786	15			

**Table A8:** Tukey test statistic and absolute difference between sample means for Young’s modulus blocked by testing temperature.

Room Temperature				
Tukey Test Statistic (GPa)		Horizontal	Vertical	Injection-Molded
0.882	Diagonal	0.0516	1.68	4.90
	Horizontal		1.63	4.85
	Vertical			3.22
40 °C				
Tukey Test Statistic (GPa)		Horizontal	Vertical	Injection-Molded
0.819	Diagonal	0.0156	1.76	4.70
	Horizontal		1.78	4.72
	Vertical			2.95
50 °C				
Tukey Test Statistic (GPa)		Horizontal	Vertical	Injection-Molded
1.34	Diagonal	0.781	1.70	6.42
	Horizontal		0.923	5.64
	Vertical			4.71
60 °C				
Tukey Test Statistic (GPa)		Horizontal	Vertical	Injection-Molded
1.92	Diagonal	1.01	1.47	4.02
	Horizontal		0.460	3.01
	Vertical			2.55
70 °C (λ = −0.620)				
Tukey Test Statistic (GPa)		Horizontal	Vertical	Injection-Molded
0.853	Diagonal	1.79	0.849	3.47
	Horizontal		0.939	1.68
	Vertical			2.62
80 °C (λ = −0.603)				
Tukey Test Statistic (GPa)		Horizontal	Vertical	Injection-Molded
0.0467	Diagonal	0.0148	0.00978	0.0445
	Horizontal		0.00497	0.0297
	Vertical			0.0347

## Appendix C. Specimen Images after Testing

Figure A1 shows the void space of an ASTM D638-14 Type V vertical specimen tested at 50 °C. Figure A2 shows the void space of an ASTM D638-14 Type V horizontal specimen tested at 50 °C. Figure A3 shows the void space of an ASTM D638-14 Type V diagonal specimen tested at 50 °C. Figure A4 shows the void space of an ASTM D638-14 Type V vertical specimen tested at room temperature. Figure A1 shows both halves of a failure (vertical tested at 50 °C).

## Appendix D. Average Strain at Different Temperatures

Table A9 shows the average strain of horizontal, vertical, and diagonal samples at all the tested temperatures.

**Table A9:** Average strain of horizontal, vertical, and diagonal samples at all tested temperatures.

Diagonal						
Temperature (°C)	Room	40	50	60	70	80
Average Strain (%)	0.74	0.74	0.67	0.98	5.78	40.25
Horizontal						
Temperature (°C)	Room	40	50	60	70	80
Average Strain (%)	1.03	1.02	1.10	1.03	6.17	35.69
Vertical						
Temperature (°C)	Room	40	50	60	70	80
Average Strain (%)	1.04	0.97	1.10	1.13	8.65	33.47
